# Impact of organized activities on mental health in children and adolescents: An umbrella review

**DOI:** 10.1016/j.pmedr.2021.101687

**Published:** 2021-12-27

**Authors:** Mirte Boelens, Michel S. Smit, Hein Raat, Wichor M. Bramer, Wilma Jansen

**Affiliations:** a Department of Public Health, Erasmus MC, University Medical Center, PO BOX 2040, 3000 CA Rotterdam, the Netherlands; b Medical Library, Erasmus MC, University Medical Center, PO BOX 2040, 3000 CA Rotterdam, the Netherlands; c Department of Social Development, Municipality of Rotterdam, PO BOX 70032, 3000 LP Rotterdam, the Netherlands

**Keywords:** WHO, World Health Organization, PRISMA-P, Preferred Reporting Items for systematic reviews and *meta*-analyses Protocol, PRISMA, Preferred Reporting Items for systematic reviews and *meta*-analyses, CCA, Corrected covered area, AMSTAR-2, A Measurement Tool to Assess Systematic Reviews, GRADE, Grading of Recommendations, Assessment, Development, and Evaluation, Depression, Anxiety, Mental health, Sport, Extracurricular, Youth

## Abstract

Mental health problems are a leading cause of health-related disability among children and adolescents. Organized activities are a possible preventive factor for mental health problems. An aggregated overview of evidence is relevant for youth policymakers and is lacking so far.

Thus we aim to provide an overview of published systematic reviews and *meta*-analyses on the impact of participation in organized sport and non-sport activities (e.g. arts, music) on childhood and adolescent mental health. Systematic reviews were identified through a search in five databases (Embase, MEDLINE, Web of Science core collection, CINAHL and PsycINFO) on 25-March-2021. Systematic reviews about organized activities and mental health outcomes in 0–21-year-olds published in English were included. Two independent reviewers assessed titles, abstracts and full texts, performed data-extraction and quality assessment using the AMSTAR-2 and assessed the quality of evidence. Out of 833 studies, six were considered eligible. Quality of the reviews ranged from critically low to moderate. Most reviews focused on organized sport activities, focusing on: team sport, level of sport involvement, extracurricular and community sport activities. Indications of a positive impact on mental health outcomes were found for participation in team sport, in (school) clubs, and in extracurricular and community sport and non-sport activities. We found a small positive impact of organized sport activities on mental health outcomes among children and adolescents. This seems not to depend on any specific type of organized sport activity. Limited evidence was found for organized non-sport activities.

## Highlights

• This umbrella review included six systematic reviews of lower quality.
• A small positive impact of organized sport activities on youth mental health was found.
• Only two systematic reviews that studied organized non-sport activities could be included.
• Mixed results were found for impact of organized non-sport activities on youth mental health.
• Mediation or confounding through physical activity needs further study.

## 1 Introduction

Mental health problems are a leading cause of health-related disability among children and adolescents (Kieling et al., 2011, Keyes, 2002). Worldwide, around 10–20% of all children and adolescents experience mental health problems (Kessler et al., 2007). Mental health problems during childhood or adolescence can also have implications in adulthood such as mental health problems or employment difficulties (Copeland et al., 2015, Fergusson et al., 2007, Ning et al., 2020). However, according to the World Health Organization (WHO) mental health is more than the absence of mental health problems. It is “a state of well-being in which the individual realizes his or her own abilities, can cope with the normal stresses of life, can work productively and is able to make a contribution to the community” (World Health Organization. Promoting mental health: concepts, emerging evidence, practice: summary report. Geneva;, 2004). In a similar vein, Keyes suggests that absence of mental health (i.e. languishing), is not similar to good mental health (i.e. flourishing) (Galderisi et al., 2015, Keyes, 2002, Keyes, 2005, Keyes, 2007, Westerhof and Keyes, 2010, King et al., 2014). Exploring possible preventive factors for reducing mental health problems and for promoting good mental health is thus important.

One possible factor for reducing mental health problems or promoting good mental health is participation in organized activities, whether sport or non-sport (Bohnert et al., 2010, Lerner et al., 2012). The positive youth development theory, grounded in the socio-ecological systems theory, postulated that sport and non-sport organized activities may offer opportunities for children and adolescents to develop relationships, engage in activities that increase their confidence, competence, character, caring and connectedness (Agans et al., 2016, Bronfenbrenner, 2003). Consequentially it is hypothesized that they are at lower risk for academic, psychological, social and behavioral problems (Bohnert et al., 2010, Lerner et al., 2012). This may depend on the type, breadth, intensity and duration of the activities (Bohnert et al., 2010).

Organized sport and non-sport activities can be defined as activities that are structured, supervised by adults, emphasize skill building, are generally voluntary, have regular scheduled meetings and are not part of the school curriculum (Bohnert et al., 2010, Mahoney et al., 2005, Mahoney et al., 2005). Examples of organized activities include but are not limited to sport, arts, music and community programs (Bohnert et al., 2010, Mahoney et al., 2005, Mahoney et al., 2005). Features of organized activities that have been found to improve mental health are: safe and appropriate peer interactions, structure and adult supervision, forming of supportive relationships with peers and adults, emphasis on inclusion and a sense of belonging, emphasis on positive social norms, support of efficacy and mattering and skill-building (Mahoney et al.,). Organized sport activities also includes physical activity as an additional feature that may improve the mental health of children and adolescents (Biddle and Asare, 2011). Organized non-sport activities may include physical activity but not always (e.g. scouting or dance). Local policies can influence and encourage participation in organized sport and non-sport activities and its determinants.

Several studies examined the impact of various types of organized sport and non-sport activities on aspects of child and adolescent mental health. These suggest a possible beneficial impact on mental health such as behavioral outcomes, self-esteem and self-confidence (Bungay and Vella-Burrows, 2013, Zarobe and Bungay, 2017). Contrary, some studies have observed harmful consequences of organized activities, such as risk behavior and bullying(Badura et al., 2017, Fredricks and Eccles, 2008, Matjasko et al., 2019, Randall and Bohnert, 2012) In two of these studies this depended on the amount of time that was spend in the organized activities(Matjasko et al., 2019, Randall and Bohnert, 2012) ()

As far as we know, there currently is no overview aggregating the available evidence from systematic reviews on the impact of participation in organized sport and non-sport activities on child and adolescent mental health, while such an overview would be highly relevant for policymakers in designing more effective preventive youth policies. Thus, the aim of this study is providing an overview of the evidence of the impact of organized sport and non-sport activities on childhood and adolescent mental health outcomes from a public health perspective based on an umbrella review of published systematic reviews.

## 2 Methods

A protocol prospectively registered within PROSPERO (CRD42020213597, available via https://www.crd.york.ac.uk/prospero/display_record.php?ID=CRD42020213597) on November 9th 2020. The protocol was developed in accordance with the Preferred Reporting Items for systematic reviews and *meta*-analyses Protocol (PRISMA-P) (Moher et al., 2015). This umbrella review adheres to the PRISMA statement and used guidance from Arotomatis et al. (Aromataris et al., 2015, Page et al., 2020).

### 2.1 Eligibility

Peer-reviewed systematic reviews with and without *meta*-analyses were considered eligible (Aromataris et al., 2015). Other types of reviews (i.e. narrative or rapid reviews) were excluded as we aim to summarize studies with the highest level of evidence (Ioannidis, 2009).

Systematic reviews containing observational study designs (i.e. case-control, cohort, cross-sectional) and trial designs in any form including pilot studies were included. Qualitative designs were considered not eligible. Systematic reviews containing both quantitative and qualitative designs were included.

Systematic reviews containing studies with children and adolescents with a mean age between 0 and 21 years old were included (Hardin et al., 2017). Systematic reviews in which any participant was aged ≥ 25 years old without sub-analysis for participants aged < 25 years old with a mean age between 0 and 21 years old, were excluded. Systematic reviews on general populations were included, as well as reviews with sub-analyses on general populations. Systematic reviews that included studies on fully clinical or at-risk populations (e.g. attention deficit hyperactivity disorder (ADHD), traumatic experiences) were excluded as we aimed to study the impact of organized activities on mental health from a public health perspective.

Systematic reviews containing organized sport or non-sport activities were included. For this umbrella-review a definition based upon the definition postulated by Bohnert et al., was used (Bohnert et al., 2010, Mahoney et al., 2005, National Research Council and Institute of Medicine, 2002). Their definition is: “Organized activities is a blanket term that refers to a broad range of adult-sponsored activities that fall outside the regular school curriculum and include diverse contexts such as school-based extracurricular activities, community organizations, and youth development programs. Despite the differences in focus, organized activities share several common features. The activities are generally voluntary, hold regularly scheduled meetings, are supervised by adults, include other participants, are organized around particular competencies, and tend to be rule-based.” (Bohnert et al., 2010, Mahoney et al., 2005, National Research Council and Institute of Medicine, 2002) In this umbrella review we broaden this definition and include organized activities without other participants (e.g. individual arts or music lessons, individual resistance training), organized activities that are not rule-based. Organized activities should be provided by a volunteering- or non-volunteering party (i.e. not organized by children or adolescents themselves).”

The control group or comparator condition is formed by children and adolescents not exposed to organized sport activities and/or non-sport activities (e.g. non– organized sport or non– sport activities, no sport or non-sport activities).

There were no limitations regarding country. Systematic reviews were included if they included organized activities occurring in an extracurricular (after-school) or community (e.g. clubs, community centers) setting corresponding with our definition. Systematic reviews focusing on sport or non-sport activities that took place within school-curricula or clinical settings were excluded.

The outcomes of interest were indicators of me mental health as defined by the WHO and by Keyes (Galderisi et al., 2015, Keyes, 2002, Keyes, 2005, Keyes, 2007, Westerhof and Keyes, 2010, King et al., 2014, World Health Organization, 2004). The WHO defined mental health as follows: “mental health is more than the absence of mental health problems. It is a state of well-being in which the individual realizes his or her own abilities, can cope with the normal stresses of life, can work productively and is able to make a contribution to the community” (World Health Organization, 2004). The definition of mental health by Keyes consists of three aspects: emotional well-being, psychological well-being and social well-being. According to Keyes absence of mental health problems (i.e. languishing) is not similar to good mental health (i.e. flourishing) and mental health should be seen as a continuum (Galderisi et al., 2015, Keyes, 2002, Keyes, 2005, Keyes, 2007, Westerhof and Keyes, 2010, King et al., 2014). In line with these definitions we did not only include all (aspects of) mental health problems as indicators of mental health and thus as outcomes of interest (e.g. anxiety, depression, ADHD and other mental health problems), but also aspects of mental well-being (e.g. self-esteem, efficacy, self-worth). No preferred outcome measure was formulated a priori. Systematic reviews that did not report on at least one aspect of mental health were excluded.

Systematic reviews published in English were included. Systematic reviews in other languages were excluded because of language barriers of the authors.

### 2.2 Search strategy

A systematic literature search was conducted in five databases (Embase.com, MEDLINE via Ovid, Web of Science core collection (See Appendix A for our core collection), CINAHL via EBSCOhost and PsycINFO via Ovid) from inception to March 25th 2021 (date last searched). The search strategy was developed by a Medical Librarian (WMB) and combined thesaurus terms as well as terms in title abstract for three elements: sport or participation, mental health or behavior, children or adolescents, and was limited to systematic reviews and *meta*-analysis. The syntax and thesaurus terms of the search strategy were adapted to each distinct database. In the search strategy, no language or date limits were applied. Appendix A includes the full search strategy. References of relevant reviews were screened for other relevant systematic reviews.

### 2.3 Selection process

Endnote X9 was used for the selection process. Duplicates of records were retrieved and removed using the method described by Bramer et al. (Bramer et al., 2016) Two independent reviewers (MB and MS) performed title and abstract screening to identify eligible reviews and subsequently performed full-text screening. Disagreements at both stages were resolved through discussion until consensus was reached, and, if necessary, resolved by consulting a third independent reviewer (WJ).

### 2.4 Data extraction

Data were extracted independently by two reviewers (MB and MS). Extracted information included: first author, year of publication, included languages of primary studies, objective, eligibility criteria, if it included a *meta*-analysis, number and type of primary studies, sample (size, age and sex), number of databases searched, range of publication date, instrument for quality appraisal and quality rating, intervention(s)/phenomena of interest, outcome(s) and outcome measure(s), measurement instruments and funding.

### 2.5 Overlap

The corrected covered area (CCA) was used to calculate the amount of overlap of primary studies included in the systematic reviews (Pieper et al., 2014). The CCA is calculated by dividing the frequency of repeated occurrences of index studies (first occurrence of primary study) in other reviews by the product of the number of index studies and the number of reviews, minus the number of reviews. The CCA can be represented as a percentage between 0 and 100%. A CCA of 0–5% is considered slight overlap, a CCA of 6–10% is considered moderate overlap, a CCA of 11–15% is considered high overlap and a CCA > 15 is considered very high overlap (Pieper et al., 2014). For the calculation, see Appendix B.

### 2.6 Risk of bias

Two reviewers (MB and MS) independently assessed risk of bias of the included systematic reviews using the A Measurement Tool to Assess Systematic Reviews (AMSTAR-2) (Shea et al., 2017). Discrepancies were resolved through discussion until consensus was reached and if necessary by consulting a third independent reviewer (WJ). The AMSTAR-2 consists of sixteen items. Seven items are considered critical, these are: a priori protocol, adequate search strategy, providing justification for excluded studies, appropriate risk of bias assessment, appropriate statistical methods in *meta*-analysis, accounting for risk of bias when interpreting results and publication bias. Three items concern *meta*-analytical methods and are not applicable for systematic reviews without *meta*-analysis. The AMSTAR-2 rates systematic reviews as: critically low (more than one critical weakness with or without non-critical weaknesses), low (one critical weakness with or without non-critical weaknesses), moderate (more than one non-critical weakness) or high quality (no or one non-critical weakness) (Shea et al., 2017).

### 2.7 Analysis

Because of the broad scope of organized activities and mental health outcomes included in this umbrella review performing a *meta*-analysis was not possible. Results were narratively (qualitative and quantitative) synthesized. Results were grouped by type of organized activities (i.e. sport, non-sport or both) and further subdivided by type of organized sport activity. As *meta*-analyses used different effect sizes or measures of association, we converted all reported effect sizes and measures of association to the Cohen’s d, for comparison purposes (Fusar-Poli and Radua, 2018, Sanchez-Meca et al., 2003). Formulas for these conversions are reported in Appendix C. For the summary of findings, quality of evidence per determinant was assessed by a self-developed decision scheme including: *meta*-analysis, number of primary studies, significance, direction, magnitude and imprecision. Scores were: no indication, mixed findings, insufficient evidence, there is an indication or high certainty. A self-developed scheme was used as suitable schemes for umbrella reviews are lacking (Appendix D).

## 3 Results

**Fig. 1:**
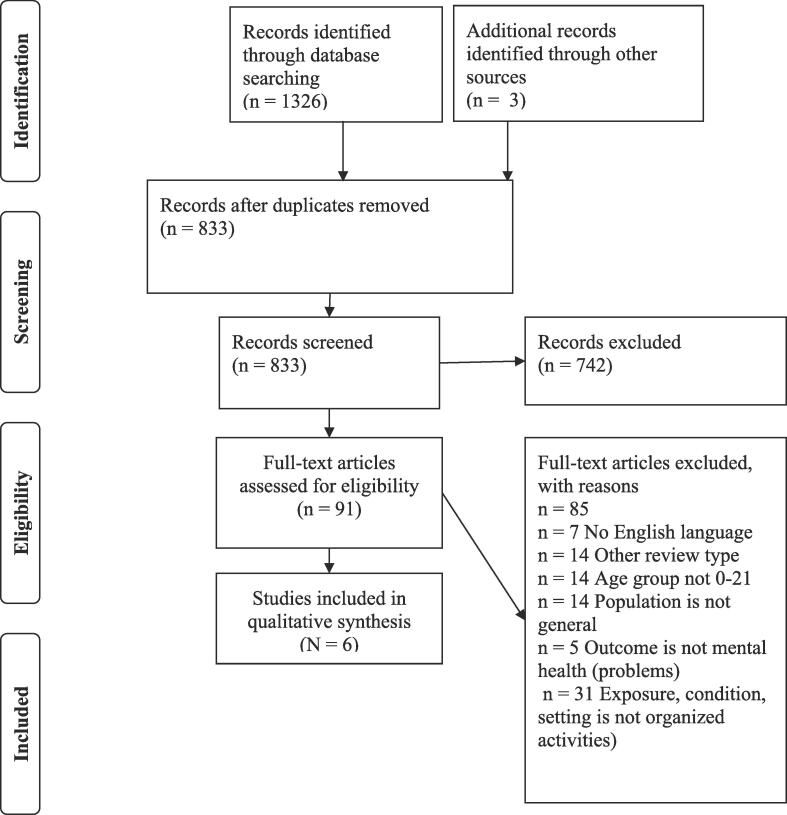
Flow diagram showing the selection process in the umbrella review.

After deduplication, 833 records remained. After all rounds of screening six systematic reviews were considered eligible (Cairns et al., 2014, Collins et al., 2019, Eime et al., 2013, Evans et al., 2017, Zuckerman et al., 2020, Panza et al., 2020). Fig. 1 describes the results of the search and study selection process. Appendix E includes references (n = 85) of all studies excluded after full-text screening subdivided by reason for exclusion.

Table 1 shows the characteristics of the six included systematic reviews. Systematic reviews were published between 2013 and 2020 (Cairns et al., 2014, Collins et al., 2019, Eime et al., 2013, Evans et al., 2017, Zuckerman et al., 2020, Panza et al., 2020). Four systematic reviews included *meta*-analyses (Cairns et al., 2014, Collins et al., 2019, Eime et al., 2013, Zuckerman et al., 2020). Sample sizes of the systematic reviews ranged from 460 to 234,503 participants. Databases searched ranged from 1 to 13. Included primary studies ranged from 7 to 113 studies. Most primary studies were from North America, followed by Europe and Australia. Five primary studies were from Asia and two from Africa. Primary studies were published between 1988 and 2020.

**Table 1:** Characteristics of included systematic reviews.

**Author & year**	**Type of review**	**Objective**	**Databases and data range of search**	**Sample size**	**Included studies (design, n)**	**Publication data range**	**Funding**
Cairns et al., 2014	SR_a_ & MA_b_	To identify risk and protective factors that are associated with depression adolescents (between 12 and 18 y_c_) with a focus on those factors that are potentially modifiable by the adolescent.	PsycINFO, PubMed, Scopus (n = 3). Inception-Sept 2013.	n = 234,503	SR: n = 113 MA: n = 69 Longitudinal (n = 113).	1986–2013	Australian Postgraduate Award from the Australian Federal Government. National Health and Medical Research Council Australia Fellowship (566652)
Collins et al., 2019	SR & MA	To investigate the effect of RT_d_ interventions on ‘the self’ in youth.	Embase, ERIC, MEDLINE, PsycINFO, PubMed, SCOPUS, SPORTDiscuss (n = 7). Inception-Oct 2018.	SR: n = 460 MA: n = 375	SR: n = 7 MA: n = 4 Pre-post design (n = 7).	SR:1988–2017 MA: 2010–2015	No
Eime et al., 2013	SR	a) To investigate the psychological and social benefits of participation in sport for children and adolescents, b) To develop a conceptual model.	AU SPORT, AusportMed, CINAHL, Cochrane Library, EBSCHOHost Research Databases, Health Collection, Informit, Medline Fulltext, PsycARTICLES, Psychology and Behavioral Sciences Collection, PsycINFO, PubMed, Scopus, SPORTDiscus Fulltext (n = 14). Jan 1990-May 2012.	n = 143,489	SR: n = 30. Longitudinal (n = 9) Cross-sectional, quantitative (n = 21; of which 2 qualitative).	1993–2011	VicHealth Research Practice Fellowship.
Evans et al., 2017	SR	To investigate the psychological and social benefits of participation in sport for children and adolescents.	CINAHL, ERIC, MEDLINE, PsycINFO, SPORTDiscus (n = 5). Jan 1980-May 2016.	n = 35,257	SR: n = 35 Longitudinal (n = 12). Cross-sectional (n = 19). Other (n = 4).	1995–2016	No
Panza et al., 2020	SR & MA	To investigate the correlation between mental health and organized sport participation among adolescents aged 12–18 y.	ERIC, MEDLINE, PsycINFO, SPORTDiscus ,Web of Science (n = 5). Inception-Oct 2018.	SR: n = 110,054 MA: unclear	SR: n = 28 MA: n = 20 Longitudinal (n = 16). Cross-sectional (n-13).	2000–2019	Unclear
Zuckerman et al., 2020	SR & MA	To assess the association between team sport participation and health outcomes in young, school-aged athletes from ages 5- to 25-years old. Health outcomes were divided into three domains: 1) behavioral, 2) psychological or 3) social.	PubMed (n = 1)	SR: n = 106,887 MA: n = 52,122	SR: n = 34 MA: n = 9 Design: unclear	1996–2020	No

**Author & year**	**Setting**	**Population characteristics: Age range, Sex (% girls), and Other**	**Continents**	**Quality assessment, assessed quality**	**Condition**	**Comparison**	**Mental health outcome(s)**	**Measurement tool(s)**
Cairns et al., 2014	Organized sport & extracurricular activities	Age: 12–18 y_mm_ Sex: unclear Other: general population.	North America n = 81, Australia n = 10, Europe n = 19, Asia n = 3.	Unclear	Extracurricular activities defined as the extent to which the adolescent is involved in activities that occur outside or parallel to the traditional school curriculum (e.g. clubs, teams, volunteering) & Sport defined as involvement in organized sport (individual or team based).	Unclear	The onset of unipolar depressive disorders as classified in the DSM-IV-R_e_	NlMH-DIS_f_, CES-D_g_, CES-DC_h_, MDI_i_, CDI_j_, self-developed.
Collins et al., 2019	Community sport & extracurricular activities	Age: 10–16 y_nn_ Sex: SR:52.5% Other: general/healthy n = 2, mixed n = 3, overweight/obese n = 2.	North America n = 5, Australia n = 2.	Quality Assessment Tool for Quantitative Studies n = 2; 28.6% regarded as high quality	RT methods, excluding studies with resistance training methods containing plyometric, vibration or neuromuscular training, training specifically for rehabilitation purposes or additional activity.	No resistance training, nutrition input only, normal activity, PE	Perceived body attractiveness, global self-worth, physical self-worth	M−ZSCSC_k_, PSE_l_, Adapted CSES_m_, CY-PSPP_n_, CY-PSPP_n_ adolescents, Exercise SEQ_o_, PSW_p_ , CY-PSPP Self-Esteem sub-scale_q_^,^ modified RT_d_ Self-efficacy, modified RT_d_ Outcome Expectancy Questionnaire, self-developed.
Eime et al., 2013	Unclear	Age: 6–12 y_nn_ Sex: unclear Other: unclear	North America n = 25, Europe n = 5.	Downs and Black tool n = 10 (33%) in the highest tertile.	Participation in sport. Sport defined as a human activity of achieving a result requiring physical exertion and/or physical skill which, by its nature and organization, is competitive and is generally accepted as being a sport (i.e. as team sport, extracurricular activity, school/club sport, level of sport involvement, sport)	No sport, other PA, less/no team sport, other EA, other OA, recreation sport participation, other structured an unstructured activities, less or no school sport, non-sport club member, non-sport participation	Risk of depression and mental ill health, developmental aspects/behavior, social anxiety and shyness, self-esteem, suicidal behavior.	unclear
Evans et al., 2017	Community sport & extracurricular sport	Age: 7–20 y_nn_ Sex: 51.5% (computed based on 33/35 studies Other: unclear	North America n = 17, Australia n = 3, Europe n = 8, Asia n = 1, Africa n = 1.	Downs and Black tool, adapted version n = 25 (71.4%) had an average risk of bias score.	Organized sport defined as competitive activity requiring skill coordination and/or exertion - generally accepted as being 'sport'. Also includes features that distinguish it as organized (e.g. Regular participation schedule, rule-guided, adult leaders, and social commitment).	Different activity types or different levels of an activity.	Psychosocial health and well-being, sport-specific or general self-concept, moral beliefs, development of positive assets, sport-specific motives and intentions and sport group environments.	Item created to assess sport enjoyment from the RSES_q_, CES-D_g_, parent-reported ESAK_r_, items assessing intentions to return from SCM_s_, WHO-5_t_, parent-reported HRQOL_u_ , social and task interdependence subscales YES_v_, Self-developed and some not reported.
Panza et al., 2020	Community sport & school-based sport	Age: 11.5–17.7 y_nn_ Sex: 52.9% Other: unclear	North America n = 19, Australia n = 3, Europe n = 6, Africa n = 1.	Downs and Black tool, adapted version n = 24 (82.8%) regarded as having a relatively low risk of bias.	Sport participation defined as a type of physical and competitive OA that is played on a team or as an individual and shaped by facilities, policies, and normative beliefs that the activity is seen as a sport.	No sport involvement or no/low frequency.	Anxiety symptoms and Depression symptoms.	BDI_w_, BDI-II_w_, CDI_j_, CES-D_g_, CES-DC_g_,CIDI_x_, DASS_y_, GHQ-12^z^, HADS_aa_, MDI_i_, PHQ-9_bb_, SCL-90(R)_cc_, SMFQ_dd_, CCHS_ee_, SCAS_ff_, STAI_gg_, Zung SAS_hh_, some not reported.
Zuckerman et al., 2020	Team sport	Age: 5–25 y_mm_ Sex: Unclear Other: Unclear	North America n = 20, Europe n = 12 (n = 1 is from Australia and Czech republic), Asia n=1.	ROBINS-I n = 32 (94%) of the studies were regarded as having a low risk of bias.	Team sport participation	No team sport participation (i.e. individual or no sport participation) or none	Behavioral, psychological and social health outcomes (such as anxiety/depressive symptoms).	Anxiety: GAD-7_ii_ and self-reported; Depression: Beck depression inventory second edition and self-reported; Self-worth: WSDQ_jj_; Psychosocial health: SDQ_kk_; Social behavior: SBI_ll_; Self-esteem: self-reported and other Anorexia nervosa: ORTO-15; Quality of Life: KIDSCREEN-52; Delinquency & life satisfaction: self-reported 7-point life satisfaction scale Social identity: self-reported twelve-item 7-point scale assessing cognitive centrality, in-group ties and in-group affect High risk activity: other

^a^SR=systematic review; _b_MA=Meta=analysis; _c_y=years; _d_RT=resistance training; _e_DSM-IV-R = Diagnostic and Statistical Manual of Mental Disorders Revised; _f_NIMHH-DIS=National Institute of Mental Health Diagnostic Interview Schedule; _g_CES-D=Center for Epidemiological Studies Depression Scale; _h_CES-DC=Center for Epidemiological Studies Depression Scale for Children; _i_MDI=Major Depression Inventory; _j_CDI=Children's Depression Inventory; _k_M-ZSCSC=The Martinek Zaichkowsky Self-concept Scale for Children; _l_PSE=Physical Self-Efficacy Scale; _m_CSES=Children’ s Self Efficacy Scale; _n_CY-PSPP=Physical Self-Perception Profile for Children and youth; _o_SEQ=Self-efficacy Scale; _p_PSW=Physical Self Worth Scale; _q_RSES=Rosenberg Self-Esteem Scale;_r_ESAK=Social Anxiety in Children and Adolescents; _s_SCM=Sport Commitment Questionnaire; _t_WHO-5=WHO-5 Well-Being Index;_u_HRQOL= Health Related Quality of Life; _v_YES=Youth Experience Survey 2.0; _w_BDI=Beck Depression Inventory;_x_CIDI=Composite International Diagnostic Interview;_y_DASS=Depression Anxiety Stress Scales;_z_GHQ-12=General Health Questionnaire-12;_aa_HADS=Hospital Anxiety and Depression Scale;^bb^PHQ-9=Patient Health Questionnaire-9; _cc_SCL-90(R)=Symptom Checklist-90-Revised;_dd_SMFQ=Short Mood and Feelings Questionnaire;_ee_CCHS=Canadian Community Health Survey;_ff_SCAS=Spence Children's Anxiety Scale;_gg_STAI=State-Trait Anxiety Inventory;_hh_Zung SAS=Zung Self-Rating Anxiety Scale;_ii_GAD-7=Generalized Anxiety Disorder;_jj_WSDQ=Washington self-description questionnaire;_kk_SDQ=Strengths and Difficulties questionnaire;_ll_SBI=Sports Behavior Inventory.

*Based on inclusion criteria. ^†^Based on included primary papers for which age was reported.

All six systematic reviews reported on organized sport activities and two also reported on organized non-sport activities (Cairns et al., 2014, Eime et al., 2013). Five systematic reviews examined individual and team-based sport (Cairns et al., 2014, Eime et al., 2013, Evans et al., 2017, Zuckerman et al., 2020, Panza et al., 2020). Three systematic reviews examined level of sport involvement (Eime et al., 2013, Evans et al., 2017, Panza et al., 2020). Two systematic reviews focused on extracurricular school and community non-sport activities and sport (Eime et al., 2013, Evans et al., 2017). One examined resistance training (Collins et al., 2019). One examined organized non-sport activities (Cairns et al., 2014). One examined (school) club sport and non-specified sport (Eime et al., 2013). One examined non-specified sport.

Table 1 shows all mental health outcomes studied in the included systematic reviews. Most studied mental health outcomes were (aspects of) mental health problems such as depressive symptoms and anxiety symptoms (Cairns et al., 2014, Eime et al., 2013, Zuckerman et al., 2020, Panza et al., 2020). Less studied were aspects of mental well-being such as development of positive assets, self-esteem, self-worth and self-concept (Collins et al., 2019, Eime et al., 2013, Evans et al., 2017).

In total 17 studies out of 118 relevant primary studies were reported in multiple systematic reviews (14.4%). Three primary studies were reported thrice in the included systematic reviews (Gore et al., 2001, Sanders et al., 2000, Zarrett et al., 2009). The CCA amounting 3.6% indicates a slight overlap. Appendix B shows the citation matrix used to calculate the overlap.

Table 2 reports the quality assessment. Five systematic reviews were identified as critically low (Cairns et al., 2014, Collins et al., 2019, Gore et al., 2001, Zuckerman et al., 2020, Panza et al., 2020). One systematic review was identified as moderate in quality (Eime et al., 2013). Common quality lowering items were lack of reporting funding in primary studies (6/6), lack of an a priori protocol (5/6), lack of a description of excluded studies (5/6). Report of funding of the primary studies is needed to assess possible bias such as changes in the design, analyses or conclusion in favor of the interests of the funder (McCrabb et al., 2021). An a priori protocol helps researchers conducting their review as it has been planned and reduces arbitrary decision-making (Moher et al., 2009). An a priori protocol also enables readers to identify deviations from the planned methods and selective outcome reporting (Moher et al., 2015). Justification for excluding studies is needed to examine the impact of their exclusion from the review (Shea et al., 2017).

**Table 2:** Quality assessment using the AMSTAR-2.

		Cairns et al., 2014	Collins et al., 2019	Eime et al., 2013	Evans et al., 2017	Panza et al., 2020	Zuckerman et al., 2020
Item 1	PICO components	No	Yes	No	Yes	No	Yes
**Item 2_a_**	A priori protocol	No	No	No	No	Partial yes	No
Item 3	Study design	Yes	No	No	No	Yes	No
**Item 4_a_**	Search strategy	Partial yes	Partial yes	Partial yes	Partial yes	Partial yes	No
Item 5	Study selection	No	Yes	No	No	Yes	Yes
Item 6	Data extraction	Yes	No	No	Yes	Yes	Yes
**Item 7_a_**	Excluded studies	No	No	No	Yes	No	No
Item 8	Description of included studies	No	No	Partial yes	Partial yes	Yes	No
**Item 9_a_**	RoB assessment	No	Partial yes	Partial yes	No	No	Yes
Item 10	Reported funding	No	No	No	No	No	No
**Item 11_a_**	Meta-analyses methods	Yes	No	NA_b_	NA_b_	Yes	Yes
Item 12	Assess impact RoB on results meta-analysis	No	Yes	NA_b_	NA_b_	No	Yes
**Item 13_a_**	Account for RoB in interpreting/discussing of results	No	Yes	Yes	Yes	No	No
Item 14	Explanation of heterogeneity	No	Yes	Yes	Yes	Yes	Yes
**Item 15_a_**	Publication bias	Yes	Yes	NA_b_	NA_b_	Yes	No
Item 16	Conflict of interest	Yes	Yes	Yes	Yes	No	Yes
**Overall score**	**Quality of the review**	**Critically low quality review**	**Critically low quality review**	**Moderate quality review**	**Critically low quality review**	**Critically low quality review**	**Critically low quality of review**

^a^Indicates a critical item on the AMSTAR-2; also shown in bold; _b_NA indicates not applicable i.e. no *meta*-analysis conducted.

Rating was as follows: high quality of review: No or one non-critical weakness, Moderate quality of review: More than one non-critical weakness, Low quality of review: One critical flaw with or without non-critical weaknesses, Critically low quality of review: More than one critical flaw with our without non-critical weaknesses.

PICO = population, intervention/exposure, control/comparator, outcome; RoB = Risk of Bias.

Table 3, Table 4 provide *meta*-analysis and qualitative results, summarized findings and the quality of evidence.

**Table 3:** Summarized findings of *meta*-analysis results and quality of evidence of included systematic reviews.

Author, year	Primary studies used (n/total)	Results	Equivalent Cohen’s D effect size	Magnitude of effects	Heterogeneity (*I*^2^)	Significant (Y/N)_a_	Summary of findings and quality of evidence
		**Sport organized activities**					
		*Team-based and individual sport*					
Cairns et al., 2014	5/113	Depressive symptoms: r = -0.046 (95%CI −0.083, −0.008)	−0.092	No or negligible	Moderate (53.1%)	Y	There is an indication of a positive impact on mental health outcomes by team sport participation.
Panza et al., 2020	14/29	Depressive symptoms: ρ = − 0.08 (95%CI − 0.10, −0.06).	−0.161	No or negligible	High (80.7%)	Y	
Panza et al., 2020	9/29	Anxiety: ρ = − 0.12 (95% CI − 0.15, −0.10).	−0.242	Small negative	Moderate (71.2%)	Y	
Zuckerman et al., 2020	5/34	Depressive symptoms/anxiety: OR = 0.59 (95%CI 0.54–0.64)	−0.291	Small negative	High 97.7%	Y	
		*Level of sport involvement (*i.e. *frequency)*					
Panza et al., 2020	12/29	Depressive symptoms: ρ = − 0.09 (95%CI − 0.11, −0.06)	−0.181	No or negligible	High (88.9%)	Y	There is an indication of a positive impact on mental health outcomes by a higher level of sport involvement.
		R*esistance training*					
Collins et al., 2019	4/7	Physical self-worth: Hedges' g = 0.319 (95%CI 0.114, 0.523)	0.319	Small positive	Small to moderate (0–44.9%^2^)	Y	There are mixed findings (small positive effects but not all significant) regarding the impact on mental health outcomes by participating in resistance training.
Collins et al., 2019	3/7	Perceived body attractiveness: Hedges' g = 0.211 (95% CI −0.031, 0.454)	0.211	Small positive	Small to moderate (0–44.9%^2^)	N	
Collins et al., 2019	3/7	Global self-esteem: Hedges' g = 0.409 (95%CI 0.149, 0.669)	0.409	Small positive	Small to moderate (0–44.9%^2^)	Y	
		**Non-sport organized activities**					
		*Extracurricular non-sport activities*					
Cairns et al., 2014	8/113	Depressive symptoms: r = -0.026 (95%CI −0.122, 0.970)	−0.052	No or negligible	High (97.4%)	N	There is no indication of an impact on a mental health outcomes by participating in extracurricular activities.

^a^Statistical significance defined as a p-value < 0.05 =significant, N = non-significant.; ^2^ No individual *I*^2^ was reported.

Reported associations and effect sizes were transformed to Cohen’s D effect sizes. The magnitude of Cohen’s D was interpreted using Cohen’s D conversion. Heterogeneity was assessed using the *I*^2^ statistic. For interpretation, *I*^2^ values of 25%, 50% and 75% were considered to indicate low, moderate and high heterogeneity. Summary of findings and quality of evidence is based on a self-developed decision scheme to assess the quality of evidence.

**Table 4:** Summarized findings of qualitative results and quality of evidence of included systematic reviews.

Author, year	Primary studies used (n/total)	Results as extracted from systematic reviews	Summary of findings and quality of evidence
**Sport organized activities**			
*Sport non-specified*			
Eime et al., 2013	4/30	There were findings that sport was associated with enhanced self-concept, lower rates of suicidal ideation (including thoughts and intentions), and with positive adjustment (e.g. social skills and self-esteem).	There is an indication of a positive impact on mental health outcomes by participating in no further specified sport activities.
		*Level of sport involvement (*e.g. *frequency, duration, intensity, early involvement)*	
Eime et al., 2013	5/30	There were findings that greater sport participation was associated with lower risk of emotional distress and with lower levels of emotional and social problems. Also moderate sport participation was associated with lower depression scores. Greater participation in formal compared to informal sport was associated with lower levels of emotional and social problems. Greater frequency in sport participation led to better feelings of well-being compared to lower frequency. Total number of sport and years involved in sport was associated with better physical appearance and physical competence. Differences between competitive or non-competitive sport were minimal.	
Evans et al., 2017	16/35	There were findings for an association of early sport involvement and amount of sport involvement with psychosocial outcomes (depression and self-esteem). There was insufficient evidence for amount of individual deliberate practice or specialization in sport due to limited research.	
Panza et al., 2020	3/29	There were findings that duration of sport participation may have a small inverse correlation with depression symptoms.	
		*Resistance training*	
Collins et al., 2019	3/7	There were findings that support a positive effect of resistance training on some constructs of ‘the self ’. There was a significant increase in total self-efficacy. No evidence for a positive effect of resistance training on self-concept.	There are mixed findings regarding the impact on mental health outcomes by participating in resistance training.
		*Team-based sport*	
Eime et al., 2013	8/30	There were findings of mental health benefits (e.g. lower general risk-taking, fewer mental and general health problems, positive associations with social acceptance and self-esteem and negative associations with depressive symptoms, social isolation and mood) by participation in team-based sport. There were also findings that it was protective against feelings of hopelessness and suicidality and that it increased life satisfaction.	There are mixed findings regarding the impact on mental health outcomes by participating in team-based sport.
Evans et al., 2017	14/35	There were findings of a positive association of participation in team-based sport to psychosocial outcomes (such as youth development experiences, moral reasoning, depression and self-esteem). Some studies reported null differences regarding depressive symptoms or anxiety.	
Zuckerman et al., 2020	23/34	The majority of studies supported a positive impact of team sport participation on many behavioral and psychological health outcomes. Additional studies found similarly positive effects such as less physical fighting.	
		*(School) club sport*	
Eime et al., 2013	5/30	There were findings of higher scores on social functioning and mental health by participating in school and club sport. There were also findings of an association with superior well-being (including being better adjusted) feeling less nervous or anxious, being more often full of energy and happy about their life, feeling sad or depressed less often, having higher body image and fewer suicidal attempts. School sport participation was associated with self-esteem. A lower frequency of mental health problems by participation in competitive sport was also found.	There is an indication of a positive impact on mental health outcomes by participating in (school) club sport.
Evans et al., 2017	2/35	There were findings of an association of extracurricular school or community sport with psychosocial outcomes.	
		*Other sport*	
Evans et al., 2017	5/35	Insufficient evidence for an association of contact sport, adult involved sport, or participation in sport that require leanness or aesthetic judgements with psychosocial outcomes.	There is insufficient evidence for an impact on mental health outcomes by participating in other categories of sport. .

		**Sport and non-sport organized activities**	
		*Extracurricular school and community non-sport activities and sport*	
Eime et al., 2013	8/30	There were findings that structured activities (sport and non-sport) led to higher positive functioning. Children participating in sport and clubs had higher social skill scores compared to children who did not participate in outside-school activities. Participation in sport and non-sport organized activities led to the greatest youth development outcomes. Sport participation led to more developmental benefits than other types of extracurricular activities but the greatest benefits were seen for sport and non-sport extracurricular activities combined. Sport participation alone and in combination with non-sport activities was associated with better health outcomes, including higher healthy self-image, lower risk of emotional distress, suicidal behavior and substance abuse. There were also findings that it led higher rates of negative peer-interaction, higher rates of self-knowledge and better emotional regulation.	There is an indication of a positive impact on mental health outcomes by participating in extracurricular and community non-sport activities and sport.

Summary of findings and quality of evidence is based on a self-developed decision scheme to assess the quality of evidence.

Five systematic reviews reported results on team-based and individual sport participation (Cairns et al., 2014, Eime et al., 2013, Evans et al., 2017, Zuckerman et al., 2020, Panza et al., 2020). Three of these reported *meta*-analysis results of the impact of team-based and individual sport participation on mental health outcomes (Cairns et al., 2014, Eime et al., 2013, Panza et al., 2020). These mental health outcomes were either depressive symptoms, anxiety or a combination (Cairns et al., 2014, Eime et al., 2013, Panza et al., 2020). Summarized, a significant positive impact on mental health outcomes was found. However, the magnitude of the effect estimate of the mental health outcomes was negligible or small (i.e. reduced anxiety/depressive symptoms). The heterogeneity was either moderate or high. Three systematic reviews reported qualitatively on the impact of team-based sport on mental health outcomes (Eime et al., 2013, Evans et al., 2017, Zuckerman et al., 2020). The qualitative synthesis of team-based sport yielded mixed findings on mental health outcomes (Eime et al., 2013, Evans et al., 2017, Zuckerman et al., 2020). The mixed findings were due to some studies that found no positive or negative impact (null effect). Many different mental health outcomes were studied in the qualitative analysis (Eime et al., 2013, Evans et al., 2017, Zuckerman et al., 2020).

Three systematic reviews reported results regarding level of sport involvement (Eime et al., 2013, Evans et al., 2017, Panza et al., 2020). Level of sport involvement included frequency, a longer period/duration of sport involvement, intensity and involvement in sport at an early age (Eime et al., 2013, Evans et al., 2017, Panza et al., 2020). One systematic review reported *meta*-analysis results of the frequency of sport involvement with depressive symptoms as mental health outcome (Panza et al., 2020). Results from this review showed that there was evidence for an impact on mental health outcomes but with a negligible magnitude of effect and high heterogeneity (Panza et al., 2020). Qualitatively, findings from three systematic reviews showed a positive impact of a higher frequency, greater intensity, a longer period/duration of sport involvement and involvement at an early age (i.e. during childhood) on mental health outcomes (Eime et al., 2013, Evans et al., 2017, Panza et al., 2020).

Two systematic reviews reported results regarding extracurricular activities (Cairns et al., 2014, Eime et al., 2013). One systematic review reported *meta*-analysis results of non-sport extracurricular activities with depressive symptoms as mental health outcome (Cairns et al., 2014). Findings showed no impact of extracurricular non-sport activities on mental health outcomes as the *meta*-analysis yielded a non-significant result (Cairns et al., 2014)*.* One systematic review reported qualitative results of extracurricular non-sport and sport school and community activities with mental health outcomes (Eime et al., 2013). Findings showed a positive impact on mental health outcomes such as, higher self-image, lower risk of emotional distress, better emotional regulation and psychosocial outcomes (Eime et al., 2013). This systematic review also reported that sport participation showed greater benefits than extracurricular non-sport activities and that a combination showed greatest benefits (Eime et al., 2013).

For resistance training, sport non-specified, (school) club sport, and other sport one systematic review reported results regarding mental health outcomes (Collins et al., 2019, Eime et al., 2013, Evans et al., 2017). Meta-analysis and qualitative findings of resistance training on mental health outcomes were mixed (Collins et al., 2019). Qualitative findings reported insufficient evidence for an impact on mental health outcomes from other sport and a positive impact on mental health outcomes from sport non-specified and (school) club sport. A small number of primary studies were included (Eime et al., 2013, Evans et al., 2017).

## 4 Discussion

This umbrella review provides a detailed overview and shows that there may be a small positive impact on mental health in children and adolescents by participating in organized sport activities. Relatively much eligible research about organized sport activities and relatively less about organized non-sport activities with mental health in children and adolescents was found.

**Fig. 2:**
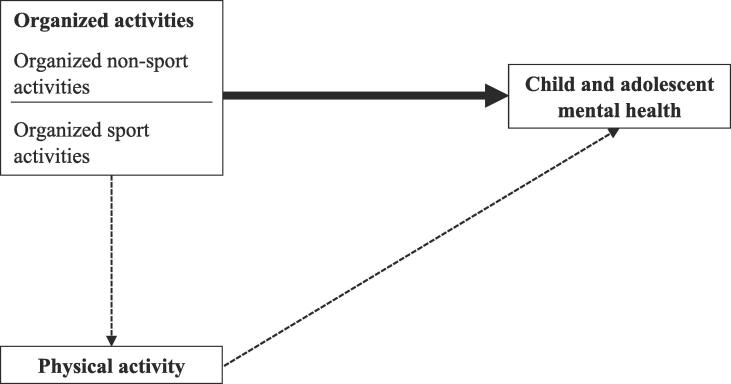
Associations of interest for this umbrella review The bold arrow indicates the impact of organized activities on child and adolescent mental health outcomes based on literature that was used for this umbrella review. The other two arrows indicate possible hypothesized pathways based on previous literature and were not studied in this umbrella review. Definitions of organized activities and mental health are given in the methods.

The objective of our umbrella review was to provide an overview of the evidence of the impact of organized sport and non-sport activities on child and adolescent mental health from a public health perspective. Fig. 2 shows the associations of interest for our umbrella review. Outside of the scope of our umbrella review and thus not studied are the association of participating in organized activities with physical activity and the association of physical activity with child and adolescent mental health. For the association of physical activity with child and adolescent mental health evidence of a positive impact was provided in the umbrella review of Biddle and Asare (Biddle and Asare, 2011). None of the systematic reviews included in our umbrella review had an objective similar to our own objective; all addressed different research questions. Cairns et al., conducted a systematic review to different modifiable risk and preventive factors associate with depression (Cairns et al., 2014). Individual and team-based sport and extracurricular activities were included as possible preventive factors and were discussed broadly (Cairns et al., 2014). Furthermore, Collins et al., and Zuckerman et al., both focused on one type of organized sport activities (i.e. resistance training and team-based sport) (Collins et al., 2019, Zuckerman et al., 2020). The other three systematic reviews focused on several types of organized sport activities and included aspects relevant for our objective such as distinguishing between different settings and patterns of involvement (Eime et al., 2013, Evans et al., 2017, Panza et al., 2020). None of the systematic reviews particularly aimed to study organized non-sport activities but in two systematic reviews it was reported (Cairns et al., 2014, Eime et al., 2013). Overall, the systematic reviews reported a positive impact of organized sport activities on mental health although some negative or null results were found. Most studied mental health outcomes were (aspects of) mental health problems.

Six systematic reviews examined the impact of organized sport activities and there may be a small positive impact on child and adolescent mental health. However, effect sizes were negligible/small and heterogeneity between studies was high thus these findings need to be interpreted with caution. According to the positive youth development theory, children and adolescents can benefit from organized activities because they can develop relationships and engage in activities increasing their confidence, competence, character, caring and connectedness (Fraser-Thomas et al., 2005, Lerner et al., 2005, Agans et al., 2016, Benson et al., 2006, Holt et al., 2020, Lerner et al., 2012). Many types of organized sport activities are included in our umbrella review and findings regarding mental health were generally in the same direction. Unfortunately, we cannot disentangle the impact of participating in organized sport activities from physical activity. None of the reviews has reported on this possible confounding or mediation, and most primary studies did not adjust for physical activity. Moreover, we cannot exclude the possibility that good mental health leads to participation in organized sport activities (Sabiston et al., 2013). Further, self-selection or drop-out could possibly also lead to a seemingly better mental health among children and adolescents who participate in organized sport activities.

The positive impact of participating in organized sport activities was hypothesized to be dependent on the breadth, intensity, and duration of participation by Bohnert et al*.* (Bohnert et al., 2010) Findings from the systematic reviews were somewhat mixed. We found evidence that children and adolescents who participated more frequently, who participated with higher intensity or who participated with longer duration (e.g. started at an early age or participated for a long period), had better mental health compared to their peers (Eime et al., 2013, Evans et al., 2017, Panza et al., 2020). However, the magnitude of the effect sizes were negligible (Eime et al., 2013, Evans et al., 2017, Panza et al., 2020).

We found no systematic review reporting exclusively on the impact of organized non-sport activities on mental health in children or adolescents. Only two systematic reviews reported on both organized sport and non-sport activities in relation to mental health among children and adolescents (Cairns et al., 2014, Eime et al., 2013). We are uncertain whether this is a gap in the literature due to scarcity of primary studies or that primary studies were not aggregated into systematic reviews. We found some systematic reviews that reported on organized non-sport activities that we excluded during the screening process. These reviews reported on yoga and mental health in clinical populations and not general populations or did not fit in our definition of organized activities, for instance because yoga was implemented as treatment (Balasubramaniam et al., 2012, Birdee et al., 2009, Cerrillo-Urbina et al., 2015, James-Palmer et al., 2020). Systematic reviews about group/choir singing and recreational rhythm music making in adults reported a positive impact on mental health outcomes (Noda et al., 2019, Williams et al., 2018, Yap et al., 2017). A recent umbrella review on performing arts, partly in the form of organized activities, in relation to many outcomes among adults also reported mixed findings regarding mental health outcomes (McCrary et al., 2021). This corresponds with the systematic reviews included in our umbrella review that yielded mixed findings on the impact of organized non-sport activities with mental (Cairns et al., 2014, Eime et al., 2013). The mixed findings could be because of a large variety in different organized non-sport activities. More systematic reviews and possibly more primary studies to examine the role of organized non-sport activities in relation to mental health in children and adolescents are needed due to the scarce available literature.

Most primary studies were performed in high-income countries. High-income countries have a large availability and variety in organized activities. Availability, affordability, transportation and cultural factors could affect participation (Mahoney et al.,). Contextual factors may influence the impact of organized activities on mental health in children and adolescents. In low and middle-income countries (LMICS) this may be different. Findings of this umbrella review may not be generalizable to LMICS. The included systematic reviews did not report differences regarding age or socioeconomic status. That no differences were reported does not imply that there are no differences. Caution is needed when generalizing these findings.

All but one systematic review received a critically low quality score by the AMSTAR-2. The AMSTAR-2 tool is stringent and rates systematic reviews as low quality in case of one critical weakness and as critically low in case of two critical weaknesses (Shea et al., 2017). Five systematic reviews have not explicitly mentioned an a priori protocol or deviated from their protocol and have not provided a detailed overview of excluded studies (with justification) at the stage of full text screening. Both critical weaknesses led to the critically low quality scores on the AMSTAR-2. If an a priori protocol or deviations were not mentioned this does not automatically mean that the study is biased, only that we do not know if it is. Providing an overview of excluded primary studies with justification shows readers that bias due to unjustified exclusion is not likely, and may increase the quality of systematic reviews. There is uncertainty regarding the bias of the systematic reviews but the results are consistent thus we feel confident about the findings. Confirmation of the reported results is needed. We emphasize the importance of adhering to the PRISMA guidelines (Page et al., 2020).

There is limited guidance on how to assess quality of evidence of outcome data in umbrella reviews (Gates et al., 2020, Pollock et al., 2016). Some umbrella reviews used the GRADE-tool (Grading of Recommendations, Assessment, Development, and Evaluation) (Pollock et al., 2016). This tool was developed for systematic reviews. For umbrella reviews the GRADE-tool could be applied by extracting the GRADE-assessment of primary studies (Pollock et al., 2016). If GRADE-assessment was not applied in the systematic reviews it is not possible to use the GRADE-tool in the umbrella-review (Pollock et al., 2016). For our umbrella review we used a self-developed decision scheme to assess the quality of evidence. We call for further development of guidance on how to assess the quality of evidence when conducting an umbrella review. Our decision scheme in the appendix might serve as a first contribution.

### 4.1 Study limitations and strengths

This umbrella review has several strengths. We included systematic reviews about a variety of organized sport activities. We used a comprehensive search strategy and two researchers independently performed the selection process, data extraction and quality assessment. We included 118 primary studies with a low degree of overlap in the included systematic reviews. This indicates no unnecessary duplication over systematic reviews (Pieper et al., 2014). Rather that the included systematic reviews complemented each other. We carefully examined the quality of the included systematic reviews and were able to elicit important issues of published systematic reviews. We used a wide definition of mental health, as our objective was to aggregate all evidence about the impact of organized activities on mental health outcomes. We used a wide age range to ensure we did not miss any studies to children and adolescents. This umbrella review also has some limitations. For this umbrella review only systematic reviews with and without *meta*-analysis were considered eligible as these types of reviews provide the highest level of evidence. Consequently, this led to the exclusion of possibly relevant systematic searches or systematized qualitative, state-of-the art, narrative, mixed methods, overviews, rapid and scoping reviews (Grant and Booth, 2009). Findings of this umbrella review are dependent on the data and that has been synthesized and reported by the included systematic reviews. Some data were lacking in the included systematic reviews. It is unclear if this is due to data lacking in primary studies. Moreover, we have not found systematic reviews that studied a negative impact of organized activities on child or adolescent mental health. We have not restricted our search to positive outcomes of mental health. That we did not find any systematic review that studied the negative impact on mental health could be due to a focus on positive mental health outcomes in previous studies and to little focus on possible negative mental health outcomes. In the included systematic reviews most studies found a positive impact or null results whereas only a few reported a negative impact. However, earlier studies have postulated that too much participating in organized activities could lead to negative outcomes on mental health such as stress, particularly among affluent children and adolescents (Larson et al., 2006, Mahoney et al., 2006, Mahoney and Vest, 2012). This is also called the over-scheduling hypothesis (Larson et al., 2006, Mahoney et al., 2006, Mahoney and Vest, 2012). Possibly, there is bias in published research which could affect the appropriateness and interpretation of our findings.

This umbrella review identified a small positive impact of organized sport activities on mental health. Although making inferences based on the included systematic reviews is difficult due to their low quality of reporting on possible bias and different mental health outcomes, the findings are consistent over the systematic reviews included. Participating in organized sport activities can be stimulated by local policy. Thus, even though the magnitude of effect sizes is small, the impact may not be small if many children and adolescents will participate in organized sport activities. Approximately 40% of children and adolescents worldwide participate in sport (Tremblay et al., 2016). Preventive policies at the local level could contribute to better mental health by stimulating more children and adolescents to participate in organized sport activities. At municipal level, this can be done by for instance increasing the amount of local sport clubs and gyms. At school level, this can be done by for instance offering additional extracurricular activities (Somerset and Hoare, 2018). Policies could focus on increasing participation in organized sport activities using for instance social media campaigns or by collaborating with local sport organizations.(World Health Organization and Office, 2016).

Further research is needed to examine whether organized activities, particularly non-sport, contribute to better mental health. Research into the impact of organized non-sport activities on child and adolescent mental health is scarce and results are mixed. Based on the positive youth development theory a positive impact on mental health could be present (Fraser-Thomas et al., 2005, Lerner et al., 2005, Agans et al., 2016, Holt et al., 2020, Lerner et al., 2012). Future research may shed light on this possible association. More high-quality primary studies and more methodologically sound systematic reviews on organized non-sport activities may ensure this. Future research should disentangle the impact of participating in organized sport activities from mere physical activity. A focus on mental well-being is also warranted in future studies to determine if organized activities have a possible impact on mental health (Galderisi et al., 2015, Keyes, 2002, Keyes, 2005, Keyes, 2007, Westerhof and Keyes, 2010, King et al., 2014, World Health Organization, 2004).

## 5 Conclusions

We found that there may be a small positive impact on mental health in children and adolescents participating in organized sport activities. This was not dependent on any specific type of organized sport activity. The observed findings should be interpreted cautiously in respect of the small effect sizes that were found, high heterogeneity of primary studies and possible publication bias. We cannot draw any conclusions about organized non-sport activities based on the small number of studies and the mixed results. Further research is needed to unravel possible mechanisms, possible mediation or confounding by physical activity and possible ways of implementing organized activities as positive preventive measure for child and adolescent mental health. This needs to be elaborated on particularly for organized non-sport activities.

## CRediT authorship contribution statement

**Mirte Boelens:** Conceptualization, Methodology, Formal analysis, Writing – original draft, Data curation, Project administration. **Michel S. Smit:** Methodology, Formal analysis, Writing – review & editing, Data curation, Project administration. **Hein Raat:** Funding acquisition, Methodology, Writing – review & editing, Supervision. **Wichor M. Bramer:** Resources, Writing – review & editing, Data curation. **Wilma Jansen:** Conceptualization, Funding acquisition, Methodology, Formal analysis, Writing – review & editing, Supervision.

## Declaration of Competing Interest

The authors declare that they have no known competing financial interests or personal relationships that could have appeared to influence the work reported in this paper.
