# Supplementary material for: Impact of organized activities on mental health in children and adolescents: An umbrella review
Source: Prev Med Rep. 2021 Dec 27;25:101687. doi: 10.1016/j.pmedr.2021.101687 (PMC8800068; doi:10.1016/j.pmedr.2021.101687)
Supplement: Supplementary data 1 [file mmc1.docx]

**Appendix A – Full search strategy per database**

| Table A1. Search per database | | | |
| --- | --- | --- | --- |
| Database searched | via | Years of coverage | Records |
| Embase | Embase.com | 1971 - Present | 467 |
| Medline ALL | Ovid | 1946 - Present | 264 |
| PsycINFO | Ovid | 1806 - Present | 137 |
| Web of Science Core Collection_a_ | Web of Knowledge | 1975 - Present | 267 |
| CINAHL | EBSCO | 1982 - Present | 191 |
| Total | | | **1326_b_** |
| _a_Science Citation Index Expanded (1975-present) ; Social Sciences Citation Index (1975-present) ; Arts & Humanities Citation Index (1975-present) ; Conference Proceedings Citation Index- Science (1990-present) ; Conference Proceedings Citation Index- Social Science & Humanities (1990-present) ; Emerging Sources Citation Index (2015-present). _b_One additional record was removed in the stage after deduplication, in total there were 776 duplicates. See also the flow- chart. | | | |

**Database 1 - embase.com**

('sport'/exp OR 'recreation'/de OR dancing/de OR leisure/de OR 'performing arts'/de OR 'recreational game'/de OR 'singing'/exp OR ('physical activity'/de AND ('social interaction'/de OR 'social participation'/de)) OR (sport* OR basketball OR baseball OR cycling OR football OR hockey OR icehockey OR fieldhockey OR jogging OR rugby OR running OR soccer OR softball OR tennis OR squash OR volleyball OR yoga OR fencing OR horse-riding OR horseback-riding OR wrestling OR judo OR karate OR jiu-jitsu OR taekwondo OR kung-fu OR martial-art* OR badminton OR padel OR water-polo OR waterpolo OR skating OR iceskating OR recreation* OR ((organi* OR participat* OR out-of-school OR outside-of-school OR leisure* OR after-school) NEAR/6 activit*) OR ((music* OR drama OR art OR singing) NEAR/6 (participat* OR activ* OR lesson* OR class))):ab,ti) AND ('mental health'/exp OR 'mental disease'/de OR 'behavior disorder'/de OR 'abnormal behavior'/exp OR 'disruptive behavior'/exp OR 'psychosocial disorder'/exp OR 'attention deficit disorder'/exp OR 'mood disorder'/exp OR 'anxiety disorder'/de OR 'adjustment disorder'/de OR 'emotional disorder'/de OR 'externalizing behavior'/de OR 'internalizing behavior'/de OR 'aggression'/de OR aggressiveness/de OR 'anger'/exp OR 'emotion regulation'/de OR 'anxiety'/de OR 'antisocial behavior'/de OR (((mental* OR psychosocial* OR psychologic*) NEAR/3 (health OR problem* OR well-being OR wellbeing OR disorder* OR disease*)) OR depress* OR ((disrupti* OR problem* OR disorder* OR disturb*) NEAR/3 (behav* OR emotion* OR mood OR adjustment*)) OR (mood NEAR/3 (change* OR swing*)) OR anxiety OR adhd OR (attention* NEAR/3 (deficit* OR disorder*)) OR hyperactiv* OR (peer* NEAR/3 (problem*)) OR ((emotional* OR social*) NEAR/3 function*) OR externali* OR internali* OR aggression* OR ((aggressive* OR antisocial* OR anti-social*) NEAR/3 behav*) OR anger OR (emotion* NEAR/3 regulation*)):ab,ti) AND (juvenile/de OR child/exp OR adolescent/exp OR adolescence/exp OR 'child behavior'/de OR 'child parent relation'/de OR pediatrics/exp OR childhood/exp OR 'child welfare'/de OR 'child development'/de OR 'child growth'/de OR 'child health'/de OR 'child health care'/exp OR 'child care'/exp OR 'child psychiatry'/de OR 'child psychology'/de OR (adolescen* OR preadolescen* OR infan* OR newborn* OR (new NEXT/1 born*) OR baby OR babies OR neonat* OR child* OR kid OR kids OR toddler* OR teen* OR boy* OR girl* OR minors OR underag* OR (under NEXT/1 (age* OR aging OR ageing)) OR juvenil* OR youth* OR kindergar* OR puber* OR pubescen* OR prepubescen* OR prepubert* OR pediatric* OR paediatric* OR school* OR preschool* OR highschool* OR suckling*):ab,ti,kw) AND ('systematic review'/de OR 'meta analysis'/de OR ((systematic* NEAR/3 review*) OR meta-analy* OR metaanaly*):ab,ti)

**Database 2 - Medline ALL Ovid**

(exp Sports/ OR Recreation/ OR Dancing/ OR Leisure Activities/ OR Games, Recreational/ OR Singing/ OR (Exercise/ AND (Interpersonal Relations/ OR Social Participation/)) OR (sport* OR basketball OR baseball OR cycling OR football OR hockey OR icehockey OR fieldhockey OR jogging OR rugby OR running OR soccer OR softball OR tennis OR squash OR volleyball OR yoga OR fencing OR horse-riding OR horseback-riding OR wrestling OR judo OR karate OR jiu-jitsu OR taekwondo OR kung-fu OR martial-art* OR badminton OR padel OR water-polo OR waterpolo OR skating OR iceskating OR recreation* OR ((organi* OR participat* OR out-of-school OR outside-of-school OR leisure* OR after-school) ADJ6 activit*) OR ((music* OR drama OR art OR singing) ADJ6 (participat* OR activ* OR lesson* OR class))).ab,ti.) AND (exp Mental Health/ OR Mental Disorders/ OR exp Social Behavior Disorders/ OR Problem Behavior/ OR exp "Attention Deficit and Disruptive Behavior Disorders"/ OR exp Mood Disorders/ OR exp Anxiety Disorders/ OR Adjustment Disorders/ OR Psychophysiologic Disorders/ OR Aggression/ OR Anger/ OR Emotional Regulation/ OR Anxiety/ OR Antisocial Personality Disorder/ OR (((mental* OR psychosocial* OR psychologic*) ADJ3 (health OR problem* OR well-being OR wellbeing OR disorder* OR disease*)) OR depress* OR ((disrupti* OR problem* OR disorder* OR disturb*) ADJ3 (behav* OR emotion* OR mood OR adjustment*)) OR (mood ADJ3 (change* OR swing*)) OR anxiety OR adhd OR (attention* ADJ3 (deficit* OR disorder*)) OR hyperactiv* OR (peer* ADJ3 (problem*)) OR ((emotional* OR social*) ADJ3 function*) OR externali* OR internali* OR aggression* OR ((aggressive* OR antisocial* OR anti-social*) ADJ3 behav*) OR anger OR (emotion* ADJ3 regulation*)).ab,ti.) AND (exp Child/ OR exp Infant/ OR exp Adolescent/ OR exp Child Behavior/ OR exp Parent Child Relations/ OR exp Pediatrics/ OR exp Child Welfare/ OR Child Development/ OR exp Child Health Services/ OR exp Child Care/ OR Child Rearing/ OR Child Psychiatry/ OR Child Psychology/ OR (adolescen* OR preadolescen* OR infan* OR newborn* OR (new ADJ born*) OR baby OR babies OR neonat* OR child* OR kid OR kids OR toddler* OR teen* OR boy* OR girl* OR minors OR underag* OR (under ADJ (age* OR aging OR ageing)) OR juvenil* OR youth* OR kindergar* OR puber* OR pubescen* OR prepubescen* OR prepubert* OR pediatric* OR paediatric* OR school* OR preschool* OR highschool* OR suckling*).ab,ti,kw.) AND (systematic review/ OR Meta-Analysis/ OR ((systematic* ADJ3 review*) OR meta-analy* OR metaanaly*).ab,ti.)

**Database 3- PsycINFO Ovid**

(exp Sports/ OR Recreation/ OR Dance/ OR Leisure Time/ OR Games/ OR Singing/ OR (Exercise/ AND (Interpersonal Relationships/ OR Interpersonal Interaction/ OR Social Interaction/ OR Participation/)) OR (sport* OR basketball OR baseball OR cycling OR football OR hockey OR icehockey OR fieldhockey OR jogging OR rugby OR running OR soccer OR softball OR tennis OR squash OR volleyball OR yoga OR fencing OR horse-riding OR horseback-riding OR wrestling OR judo OR karate OR jiu-jitsu OR taekwondo OR kung-fu OR martial-art* OR badminton OR padel OR water-polo OR waterpolo OR skating OR iceskating OR recreation* OR ((organi* OR participat* OR out-of-school OR outside-of-school OR leisure* OR after-school) ADJ6 activit*) OR ((music* OR drama OR art OR singing) ADJ6 (participat* OR activ* OR lesson* OR class))).ab,ti.) AND (exp Mental Health/ OR Mental Disorders/ OR exp Disruptive Behavior Disorders/ OR Behavior Problems/ OR exp Affective Disorders/ OR exp Anxiety Disorders/ OR Adjustment Disorders/ OR Somatoform Disorders/ OR Aggressive Behavior/ OR Aggressiveness/ OR Anger/ OR Emotional Regulation/ OR Anxiety/ OR Antisocial Personality Disorder/ OR (((mental* OR psychosocial* OR psychologic*) ADJ3 (health OR problem* OR well-being OR wellbeing OR disorder* OR disease*)) OR depress* OR ((disrupti* OR problem* OR disorder* OR disturb*) ADJ3 (behav* OR emotion* OR mood OR adjustment*)) OR (mood ADJ3 (change* OR swing*)) OR anxiety OR adhd OR (attention* ADJ3 (deficit* OR disorder*)) OR hyperactiv* OR (peer* ADJ3 (problem*)) OR ((emotional* OR social*) ADJ3 function*) OR externali* OR internali* OR aggression* OR ((aggressive* OR antisocial* OR anti-social*) ADJ3 behav*) OR anger OR (emotion* ADJ3 regulation*)).ab,ti.) AND (exp Child Behavior/ OR exp Parent Child Relations/ OR exp Pediatrics/ OR exp Child Welfare OR exp Child Care/ OR Child Psychiatry/ OR Child Psychology/ OR (adolescen* OR preadolescen* OR infan* OR newborn* OR (new ADJ born*) OR baby OR babies OR neonat* OR child* OR kid OR kids OR toddler* OR teen* OR boy* OR girl* OR minors OR underag* OR (under ADJ (age* OR aging OR ageing)) OR juvenil* OR youth* OR kindergar* OR puber* OR pubescen* OR prepubescen* OR prepubert* OR pediatric* OR paediatric* OR school* OR preschool* OR highschool* OR suckling*).ab,ti. Or 100.ag. OR 200.ag.) AND (systematic review/ OR Meta Analysis/ OR ((systematic* ADJ3 review*) OR meta-analy* OR metaanaly*).ab,ti.)

**Database 4 - Web of Science Core Collection**

(TI=(sport* OR basketball OR baseball OR cycling OR football OR hockey OR icehockey OR fieldhockey OR jogging OR rugby OR running OR soccer OR softball OR tennis OR squash OR volleyball OR yoga OR fencing OR horse-riding OR horseback-riding OR wrestling OR judo OR karate OR jiu-jitsu OR taekwondo OR kung-fu OR martial-art* OR badminton OR padel OR water-polo OR waterpolo OR skating OR iceskating OR recreation* OR ((organi* OR participat* OR out-of-school OR outside-of-school OR leisure* OR after-school) NEAR/5 activit*) OR ((music* OR drama OR art OR singing) NEAR/5 (participat* OR activ* OR lesson* OR class))) OR AB=(sport* OR basketball OR baseball OR cycling OR football OR hockey OR icehockey OR fieldhockey OR jogging OR rugby OR running OR soccer OR softball OR tennis OR squash OR volleyball OR yoga OR fencing OR horse-riding OR horseback-riding OR wrestling OR judo OR karate OR jiu-jitsu OR taekwondo OR kung-fu OR martial-art* OR badminton OR padel OR water-polo OR waterpolo OR skating OR iceskating OR recreation* OR ((organi* OR participat* OR out-of-school OR outside-of-school OR leisure* OR after-school) NEAR/5 activit*) OR ((music* OR drama OR art OR singing) NEAR/5 (participat* OR activ* OR lesson* OR class)))) AND TS=((((mental* OR psychosocial* OR psychologic*) NEAR/2 (health OR problem* OR well-being OR wellbeing OR disorder* OR disease*)) OR depress* OR ((disrupti* OR problem* OR disorder* OR disturb*) NEAR/2 (behav* OR emotion* OR mood OR adjustment*)) OR (mood NEAR/2 (change* OR swing*)) OR anxiety OR adhd OR (attention* NEAR/2 (deficit* OR disorder*)) OR hyperactiv* OR (peer* NEAR/2 (problem*)) OR ((emotional* OR social*) NEAR/2 function*) OR externali* OR internali* OR aggression* OR ((aggressive* OR antisocial* OR anti-social*) NEAR/2 behav*) OR anger OR (emotion* NEAR/2 regulation*))) AND TS=((adolescen* OR preadolescen* OR infan* OR newborn* OR (new NEAR/1 born*) OR baby OR babies OR neonat* OR child* OR kid OR kids OR toddler* OR teen* OR boy* OR girl* OR minors OR underag* OR (under NEAR/1 (age* OR aging OR ageing)) OR juvenil* OR youth* OR kindergar* OR puber* OR pubescen* OR prepubescen* OR prepubert* OR pediatric* OR paediatric* OR school* OR preschool* OR highschool* OR suckling*)) AND TI=(((systematic* NEAR/2 review*) OR meta-analy* OR metaanaly*))

**Database 5- CINAHL EBSChost**

(MH Sports+ OR MH Recreation OR MH Dancing OR MH Leisure Activities OR MH Games OR MH Singing OR MH Sporting Events OR (MH Exercise AND (MH Interpersonal Relations OR MH Social Participation)) OR TI (sport* OR basketball OR baseball OR cycling OR football OR hockey OR icehockey OR fieldhockey OR jogging OR rugby OR running OR soccer OR softball OR tennis OR squash OR volleyball OR yoga OR fencing OR horse-riding OR horseback-riding OR wrestling OR judo OR karate OR jiu-jitsu OR taekwondo OR kung-fu OR martial-art* OR badminton OR padel OR water-polo OR waterpolo OR skating OR iceskating OR recreation* OR ((organi* OR participat* OR out-of-school OR outside-of-school OR leisure* OR after-school) N5 activit*) OR ((music* OR drama OR art OR singing) N5 (participat* OR activ* OR lesson* OR class))) OR AB (sport* OR basketball OR baseball OR cycling OR football OR hockey OR icehockey OR fieldhockey OR jogging OR rugby OR running OR soccer OR softball OR tennis OR squash OR volleyball OR yoga OR fencing OR horse-riding OR horseback-riding OR wrestling OR judo OR karate OR jiu-jitsu OR taekwondo OR kung-fu OR martial-art* OR badminton OR padel OR water-polo OR waterpolo OR skating OR iceskating OR recreation* OR ((organi* OR participat* OR out-of-school OR outside-of-school OR leisure* OR after-school) N5 activit*) OR ((music* OR drama OR art OR singing) N5 (participat* OR activ* OR lesson* OR class)))) AND (MH Mental Health+ OR MH Mental Disorders OR MH Social Behavior Disorders+ OR MH Disruptive Behavior OR MH Attention Deficit Hyperactivity Disorder OR MH Affective Disorders+ OR MH Anxiety Disorders+ OR Adjustment Disorders+ OR MH Psychophysiologic Disorders OR MH Aggression OR MH Anger OR MH Emotional Regulation OR MH Anxiety OR MH Antisocial Personality Disorder OR TI (((mental* OR psychosocial* OR psychologic*) N2 (health OR problem* OR well-being OR wellbeing OR disorder* OR disease*)) OR depress* OR ((disrupti* OR problem* OR disorder* OR disturb*) N2 (behav* OR emotion* OR mood OR Nustment*)) OR (mood N2 (change* OR swing*)) OR anxiety OR adhd OR (attention* N2 (deficit* OR disorder*)) OR hyperactiv* OR (peer* N2 (problem*)) OR ((emotional* OR social*) N2 function*) OR externali* OR internali* OR aggression* OR ((aggressive* OR antisocial* OR anti-social*) N2 behav*) OR anger OR (emotion* N2 regulation*)) OR AB (((mental* OR psychosocial* OR psychologic*) N2 (health OR problem* OR well-being OR wellbeing OR disorder* OR disease*)) OR depress* OR ((disrupti* OR problem* OR disorder* OR disturb*) N2 (behav* OR emotion* OR mood OR Nustment*)) OR (mood N2 (change* OR swing*)) OR anxiety OR adhd OR (attention* N2 (deficit* OR disorder*)) OR hyperactiv* OR (peer* N2 (problem*)) OR ((emotional* OR social*) N2 function*) OR externali* OR internali* OR aggression* OR ((aggressive* OR antisocial* OR anti-social*) N2 behav*) OR anger OR (emotion* N2 regulation*))) AND (MH Child+ OR MH Infant+ OR MH Adolescent+ OR MH Child Behavior+ OR MH Parent Child Relations+ OR MH Pediatrics+ OR MH Child Welfare+ OR Child Development+ OR MH Child Health Services+ OR MH Child Care+ OR MH Child Rearing+ OR MH Child Psychiatry+ OR MH Child Psychology+ OR TI (adolescen* OR preadolescen* OR infan* OR newborn* OR (new N1 born*) OR baby OR babies OR neonat* OR child* OR kid OR kids OR toddler* OR teen* OR boy* OR girl* OR minors OR underag* OR (under N1 (age* OR aging OR ageing)) OR juvenil* OR youth* OR kindergar* OR puber* OR pubescen* OR prepubescen* OR prepubert* OR pediatric* OR paediatric* OR school* OR preschool* OR highschool* OR suckling*) OR AB (adolescen* OR preadolescen* OR infan* OR newborn* OR (new N1 born*) OR baby OR babies OR neonat* OR child* OR kid OR kids OR toddler* OR teen* OR boy* OR girl* OR minors OR underag* OR (under N1 (age* OR aging OR ageing)) OR juvenil* OR youth* OR kindergar* OR puber* OR pubescen* OR prepubescen* OR prepubert* OR pediatric* OR paediatric* OR school* OR preschool* OR highschool* OR suckling*)) AND (MH systematic review+ OR MH Meta-Analysis+ OR TI ((systematic* N2 review*) OR meta-analy* OR metaanaly*) OR AB ((systematic* N2 review*) OR meta-analy* OR metaanaly*))
