# Supplementary material for: Impact of organized activities on mental health in children and adolescents: An umbrella review
Source: Prev Med Rep. 2021 Dec 27;25:101687. doi: 10.1016/j.pmedr.2021.101687 (PMC8800068; doi:10.1016/j.pmedr.2021.101687)
Supplement: Supplementary data 2 [file mmc2.docx]

**Appendix B – Overlap of primary studies**

| Table B1. Overlap of primary studies included in the systematic reviews | | | | | | | |
| --- | --- | --- | --- | --- | --- | --- | --- |
| Number of the primary study | Unique primary study | Unique primary study is included in the systematic review | | | | | |
|  |  | **Cairns, 2014** | **Collins, 2015** | **Eime, 2013** | **Evans, 2017** | **Panza, 2020** | **Zuckerman, 2020** |
| 1 | Adachi PJC, Willoughby T. It’s Not How Much You Play, but How Much You Enjoy the Game: The Longitudinal Associations Between Adolescents’ Self-Esteem and the Frequency Versus Enjoyment of Involvement in Sports. *Journal of Youth and Adolescence*. 2014/01/01 2014;43(1):137-145. doi:10.1007/s10964-013-9988-3 |  |  |  | X |  |  |
| 2 | Agans JP, Geldhof GJ. Trajectories of Participation in Athletics and Positive Youth Development: The Influence of Sport Type. doi: 10.1080/10888691.2012.697792. *Applied Developmental Science*. 2012/07/01 2012;16(3):151-165. doi:10.1080/10888691.2012.697792 |  |  |  | X | X |  |
| 3 | Ashdown-Franks G, Sabiston CM, Solomon-Krakus S, O'Loughlin JL. Sport participation in high school and anxiety symptoms in young adulthood. *Mental Health and Physical Activity*. 2017/03/01/ 2017;12:19-24. doi:https://doi.org/10.1016/j.mhpa.2016.12.001 |  |  |  |  | X |  |
| 4 | Babiss LA, Gangwisch JE. Sports participation as a protective factor against depression and suicidal ideation in adolescents as mediated by self-esteem and social support. *J Dev Behav Pediatr*. Oct 2009;30(5):376-84. |  |  |  |  | X |  |
| 5 | Badura P, Geckova AM, Sigmundova D, van Dijk JP, Reijneveld SA. When children play, they feel better: organized activity participation and health in adolescents. *BMC Public Health*. 2015/10/24 2015;15(1):1090. doi:10.1186/s12889-015-2427-5 |  |  |  |  |  | X |
| 6 | Baldursdottir B, Valdimarsdottir HB, Krettek A, Gylfason HF, Sigfusdottir ID. Age-related differences in physical activity and depressive symptoms among 10−19-year-old adolescents: A population based study. *Psychology of Sport and Exercise*. 2017/01/01/ 2017;28:91-99. doi:https://doi.org/10.1016/j.psychsport.2016.10.007 |  |  |  |  | X |  |
| 7 | Barber BL, Eccles JS, Stone MR. Whatever Happened to the Jock, the Brain, and the Princess?: Young Adult Pathways Linked to Adolescent Activity Involvement and Social Identity. doi: 10.1177/0743558401165002. *Journal of Adolescent Research*. 2001/09/01 2001;16(5):429-455. doi:10.1177/0743558401165002 | X |  | X |  |  |  |
| 8 | Bartko WT, Eccles JS. Adolescent Participation in Structured and Unstructured Activities: A Person-Oriented Analysis. *Journal of Youth and Adolescence*. 2003/08/01 2003;32(4):233-241. doi:10.1023/a:1023056425648 |  |  | X |  |  |  |
| 9 | Begg DJ, Langley JD, Moffitt T, Marshall SW. Sport and delinquency: an examination of the deterrence hypothesis in a longitudinal study. *Br J Sports Med*. 1996;30(4):335-341. doi:10.1136/bjsm.30.4.335 |  |  |  |  |  | X |
| 10 | Boone EM, Leadbeater BJ. Game On: Diminishing Risks for Depressive Symptoms in Early Adolescence Through Positive Involvement in Team Sports. https://doi.org/10.1111/j.1532-7795.2006.00122.x. *Journal of Research on Adolescence*. 2006/03/01 2006;16(1):79-90. doi:https://doi.org/10.1111/j.1532-7795.2006.00122.x |  |  | X |  | X |  |
| 11 | Bowker A. The relationship between sports participation and self-esteem during early adolescence. doi:10.1037/cjbs2006009. *Canadian Journal of Behavioural Science / Revue canadienne des sciences du comportement*. 2006;38(3):214-229. doi:10.1037/cjbs2006009 |  |  | X |  |  |  |
| 12 | Brettschneider W-d. Effects of sport club activities on adolescent development in Germany. doi: 10.1080/17461390100071201. *European Journal of Sport Science*. 2001/06/01 2001;1(2):1-11. doi:10.1080/17461390100071201 |  |  | X |  |  |  |
| 13 | Brière FN, Yale-Soulière G, Gonzalez-Sicilia D, et al. Prospective associations between sport participation and psychological adjustment in adolescents. *Journal of Epidemiology and Community Health*. 2018;72(7):575. doi:10.1136/jech-2017-209656 |  |  |  |  | X | X |
| 14 | Bruner MW, Hall J, Côté J. Influence of sport type and interdependence on the developmental experiences of youth male athletes. doi: 10.1080/17461391.2010.499969. *European Journal of Sport Science*. 2011/03/01 2011;11(2):131-142. doi:10.1080/17461391.2010.499969 |  |  |  | X |  |  |
| 15 | Brunet J, Sabiston CM, Chaiton M, et al. The association between past and current physical activity and depressive symptoms in young adults: a 10-year prospective study. *Ann Epidemiol*. Jan 2013;23(1):25-30. | X |  |  |  | X |  |
| 16 | Calmeiro L, Stoll S, Davis PJ. Moral Reasoning in Sport: Validation of the Portuguese Version of the RSBH Value-Judgement Inventory in Adolescents. *Sport Science Review*. 2015;24:285 - 304. |  |  |  | X |  |  |
| 17 | Chen SY, Lu L. After-school time use in Taiwan: effects on educational achievement and well-being. *Adolescence*. Winter 2009;44(176):891-909. | X |  |  |  |  |  |
| 18 | Choi WS, Patten CA, Gillin JC, Kaplan RM, Pierce JP. Cigarette smoking predicts development of depressive symptoms among U.S. adolescents. *Ann Behav Med*. Winter 1997;19(1):42-50. | X |  |  |  |  |  |
| 19 | Clifford T, Blyth C. A pilot study comparing the prevalence of orthorexia nervosa in regular students and those in University sports teams. *Eat Weight Disord*. Jun 2019;24(3):473-480. |  |  |  |  |  | X |
| 20 | Darling N. Participation in Extracurricular Activities and Adolescent Adjustment: Cross-Sectional and Longitudinal Findings. *Journal of Youth and Adolescence*. 2005/10/01 2005;34(5):493-505. doi:10.1007/s10964-005-7266-8 | X |  |  |  |  |  |
| 21 | De Meester A, Aelterman N, Cardon G, De Bourdeaudhuij I, Haerens L. Extracurricular school-based sports as a motivating vehicle for sports participation in youth: a cross-sectional study. *International Journal of Behavioral Nutrition and Physical Activity*. 2014/04/07 2014;11(1):48. doi:10.1186/1479-5868-11-48 |  |  |  | X |  |  |
| 22 | Denault A-S, Poulin F, Pedersen S. Intensity of Participation in Organized Youth Activities During the High School Years: Longitudinal Associations With Adjustment. doi: 10.1080/10888690902801459. *Applied Developmental Science*. 2009/04/16 2009;13(2):74-87. doi:10.1080/10888690902801459 | X |  |  |  | X |  |
| 23 | Dimech AS, Seiler R. Extra-curricular sport participation: A potential buffer against social anxiety symptoms in primary school childre” *Psychology of Sport and Exercise*. 2011;12:347-354. |  |  | X | X |  |  |
| 24 | Dishman RK, Hales DP, Pfeiffer KA, et al. Physical self-concept and self-esteem mediate cross-sectional relations of physical activity and sport participation with depression symptoms among adolescent girls. *Health Psychol*. May 2006;25(3):396-407. |  |  |  |  | X |  |
| 25 | 1Dolenc P. Anxiety, Self-Esteem and Coping with Stress in Secondary School Students in Relation to Involvement in Organized Sports. *Zdr Varst*. Sep 2015;54(3):222-9. |  |  |  |  | X |  |
| 26 | Donaldson SJ, Ronan KR. The effects of sports participation on young adolescents' emotional well-being. *Adolescence*. Summer 2006;41(162):369-89. |  |  | X |  |  |  |
| 27 | Donkers JL, Martin LJ, Evans MB. Psychological collectivism in youth athletes on individual sport teams. *International Journal of Sport and Exercise Psychology*. 2018;16(3):285-299. |  |  |  | X |  |  |
| 28 | Doré I, O'Loughlin JL, Schnitzer ME, Datta GD, Fournier L. The longitudinal association between the context of physical activity and mental health in early adulthood. *Mental Health and Physical Activity*. 2018/03/01/ 2018;14:121-130. doi:https://doi.org/10.1016/j.mhpa.2018.04.001 |  |  |  |  | X |  |
| 29 | Dugas E, Low NC, Rodriguez D, et al. Early predictors of suicidal ideation in young adults. *Can J Psychiatry*. Jul 2012;57(7):429-36. |  |  |  |  |  | X |
| 30 | Duncan SC, Strycker LA, Chaumeton NR. Sports Participation and Positive Correlates in African American, Latino, and White Girls. doi: 10.1080/10888691.2015.1020156. *Applied Developmental Science*. 2015/10/02 2015;19(4):206-216. doi:10.1080/10888691.2015.1020156 |  |  |  |  | X |  |
| 31 | Easterlin MC, Chung PJ, Leng M, Dudovitz R. Association of Team Sports Participation With Long-term Mental Health Outcomes Among Individuals Exposed to Adverse Childhood Experiences. *JAMA Pediatrics*. 2019;173(7):681-688. doi:10.1001/jamapediatrics.2019.1212 |  |  |  |  | X | X |
| 32 | Erkut S, Tracy AJ. Predicting Adolescent Self-Esteem From Participation in School Sports Among Latino Subgroups. *Hisp J Behav Sci*. 2002;24(4):409-429. doi:10.1177/0739986302238212 |  |  | X |  |  |  |
| 33 | Faigenbaum A, Zaichowsky LD, Westcott WL, et al. Psychological effects of strength training on children. Article. *Journal of Sport Behavior*. 1997/06// 1997;20(2):164+. |  | X |  |  |  |  |
| 34 | Fatiregun AA, Kumapayi TE. Prevalence and correlates of depressive symptoms among in-school adolescents in a rural district in southwest Nigeria. *Journal of Adolescence*. 2014/02/01/ 2014;37(2):197-203. doi:https://doi.org/10.1016/j.adolescence.2013.12.003 |  |  |  |  | X |  |
| 35 | Feldman AF. *Links between school-based extracurricular activity participation and adolescent development*. 2003. | X |  |  |  |  |  |
| 36 | Ferron C, Narring F, Cauderay M, Michaud PA. Sport activity in adolescence: associations with health perceptions and experimental behaviours. *Health Educ Res*. Apr 1999;14(2):225-33. |  |  | X |  |  |  |
| 37 | Findlay L, Coplan R. Come out and play: Shyness in childhood and the benefits of organized sports participation. *Canadian Journal of Behavioural Science*. 2008;40:153-161. |  |  | X |  |  |  |
| 38 | Forsman H, Blomqvist M, Davids K, Konttinen N, Liukkonen J. The role of sport-specific play and practice during childhood in the development of adolescent Finnish team sport athletes. doi: 10.1177/1747954115624816. *International Journal of Sports Science & Coaching*. 2016/02/01 2016;11(1):69-77. doi:10.1177/1747954115624816 |  |  |  | X |  |  |
| 39 | Fredricks JA, Eccles JS. Is extracurricular participation associated with beneficial outcomes? Concurrent and longitudinal relations. *Dev Psychol*. Jul 2006;42(4):698-713. | X |  |  |  | X |  |
| 40 | Fredricks JA, Eccles JS. Participation in Extracurricular Activities in the Middle School Years: Are There Developmental Benefits for African American and European American Youth? *Journal of Youth and Adolescence*. 2008/10/01 2008;37(9):1029-1043. doi:10.1007/s10964-008-9309-4 | X |  |  |  |  |  |
| 41 | Fredricks JA, Eccles JS. Breadth of Extracurricular Participation and Adolescent Adjustment Among African-American and European-American Youth. https://doi.org/10.1111/j.1532-7795.2009.00627.x. *Journal of Research on Adolescence*. 2010/06/01 2010;20(2):307-333. doi:https://doi.org/10.1111/j.1532-7795.2009.00627.x | X |  |  |  |  |  |
| 42 | Goldfield GS, Kenny GP, Alberga AS, et al. Effects of aerobic training, resistance training, or both on psychological health in adolescents with obesity: The HEARTY randomized controlled trial. *J Consult Clin Psychol*. Dec 2015;83(6):1123-35. |  | X |  |  |  |  |
| 43 | Gomez-Baya D, Mendoza R, Matos MGd, Tomico A. Sport participation, body satisfaction and depressive symptoms in adolescence: a moderated-mediation analysis of gender differences. doi: 10.1080/17405629.2017.1364988. *European Journal of Developmental Psychology*. 2019/03/04 2019;16(2):183-197. doi:10.1080/17405629.2017.1364988 |  |  |  |  | X |  |
| 44 | Gore S, Farrell F, Gordon J. Sports Involvement as Protection against Depressed Mood. https://doi.org/10.1111/1532-7795.00006. *Journal of Research on Adolescence*. 2001/03/01 2001;11(1):119-130. doi:https://doi.org/10.1111/1532-7795.00006 |  |  | X | X | X |  |
| 45 | Gucciardi DF. The relationship between developmental experiences and mental toughness in adolescent cricketers. *J Sport Exerc Psychol*. Jun 2011;33(3):370-93. |  |  |  | X |  |  |
| 46 | Guddal MH, Stensland S, Småstuen MC, Johnsen MB, Zwart JA, Storheim K. Physical activity and sport participation among adolescents: associations with mental health in different age groups. Results from the Young-HUNT study: a cross-sectional survey. *BMJ Open*. Sep 4 2019;9(9):e028555. |  |  |  |  |  | X |
| 47 | Hammond T, Gialloreto C, Kubas H, Hap Davis Ht. The prevalence of failure-based depression among elite athletes. *Clin J Sport Med*. Jul 2013;23(4):273-7. |  |  |  |  |  | X |
| 48 | Hansen DM, Larson RW, Dworkin JB. What Adolescents Learn in Organized Youth Activities: A Survey of Self-Reported Developmental Experiences. https://doi.org/10.1111/1532-7795.1301006. *Journal of Research on Adolescence*. 2003/03/01 2003;13(1):25-55. doi:https://doi.org/10.1111/1532-7795.1301006 |  |  | X |  |  |  |
| 49 | Harrison PA, Narayan G. Differences in behavior, psychological factors, and environmental factors associated with participation in school sports and other activities in adolescence. *J Sch Health*. Mar 2003;73(3):113-20. |  |  | X |  |  | X |
| 50 | He JP, Paksarian D, Merikangas KR. Physical Activity and Mental Disorder Among Adolescents in the United States. *J Adolesc Health*. Nov 2018;63(5):628-635. |  |  |  |  | X |  |
| 51 | Hendry DT, Crocker PR, Hodges NJ. Practice and play as determinants of self-determined motivation in youth soccer players. *J Sports Sci*. 2014;32(11):1091-9. |  |  |  | X |  |  |
| 52 | Holloway JB, Beuter A, Duda JL. Self-Efficacy and Training for Strength in Adolescent Girls1. https://doi.org/10.1111/j.1559-1816.1988.tb00046.x. *Journal of Applied Social Psychology*. 1988/06/01 1988;18(8):699-719. doi:https://doi.org/10.1111/j.1559-1816.1988.tb00046.x |  | X |  |  |  |  |
| 53 | Holt NL, Kingsley BC, Tink LN, Scherer J. Benefits and challenges associated with sport participation by children and parents from low-income families. *Psychology of Sport and Exercise*. 2011/09/01/ 2011;12(5):490-499. doi:https://doi.org/10.1016/j.psychsport.2011.05.007 |  |  | X |  |  |  |
| 54 | Howie LD, Lukacs SL, Pastor PN, Reuben CA, Mendola P. Participation in activities outside of school hours in relation to problem behavior and social skills in middle childhood. *J Sch Health*. Mar 2010;80(3):119-25. |  |  | X |  |  |  |
| 55 | Howie, 2016_a_ |  |  |  |  | X |  |
| 56 | Hume C, Timperio A, Veitch J, Salmon J, Crawford D, Ball K. Physical activity, sedentary behaviour and depressive symptoms among adolescents. *Journal of Science and Medicine in Sport*. 2010/01/01/ 2010;12:e142. doi:https://doi.org/10.1016/j.jsams.2009.10.297 | X |  |  |  | X |  |
| 57 | Imtiaz F, Hancock DJ, Côté J. Examining Young Recreational Male Soccer Players' Experience in Adult- and Peer-Led Structures. *Res Q Exerc Sport*. Sep 2016;87(3):295-304. |  |  |  | X |  |  |
| 58 | Jewett R, Sabiston CM, Brunet J, O'Loughlin EK, Scarapicchia T, O'Loughlin J. School Sport Participation During Adolescence and Mental Health in Early Adulthood. *Journal of Adolescent Health*. 2014/11/01/ 2014;55(5):640-644. doi:https://doi.org/10.1016/j.jadohealth.2014.04.018 |  |  |  |  | X |  |
| 59 | Karr TM, Davidson D, Bryant FB, Balague G, Bohnert AM. Sport type and interpersonal and intrapersonal predictors of body dissatisfaction in high school female sport participants. *Body image*. 2013;10(2):210-219. |  |  |  | X |  |  |
| 60 | Kleppang AL, Hartz I, Thurston M, Hagquist C. The association between physical activity and symptoms of depression in different contexts – a cross-sectional study of Norwegian adolescents. *BMC Public Health*. 2018/12/12 2018;18(1):1368. doi:10.1186/s12889-018-6257-0 |  |  |  |  |  | X |
| 61 | Koh KT, Wang CKJ. Gender and type of sport differences on perceived coaching behaviours, achievement goal orientations and life aspirations of youth Olympic games Singaporean athletes. doi: 10.1080/1612197X.2014.932820. *International Journal of Sport and Exercise Psychology*. 2015/04/03 2015;13(2):91-103. doi:10.1080/1612197x.2014.932820 |  |  |  | X |  |  |
| 62 | Kokotailo PK, Henry BC, Koscik RE, Fleming MF, Landry GL. Substance use and other health risk behaviors in collegiate athletes. *Clin J Sport Med*. Jul 1996;6(3):183-9. |  |  |  |  |  | X |
| 63 | Kremer P, Elshaug C, Leslie E, Toumbourou JW, Patton GC, Williams J. Physical activity, leisure-time screen use and depression among children and young adolescents. *J Sci Med Sport*. Mar 2014;17(2):183-7. |  |  |  |  | X |  |
| 64 | Lee M, Whitehead J, Balchin N. The Measurement of Values in Youth Sport: Development of the Youth Sport Values Questionnaire. *Journal of Sport & Exercise Psychology*. 2000;22:307-326. |  |  |  | X |  |  |
| 65 | Lee MJ, Whitehead J, Ntoumanis N. Development of the Attitudes to Moral Decision-making in Youth Sport Questionnaire (AMDYSQ). *Psychology of Sport and Exercise*. 2007/05/01/ 2007;8(3):369-392. doi:https://doi.org/10.1016/j.psychsport.2006.12.002 |  |  |  | X |  |  |
| 66 | Leapetswe M. Goal Orientations, Sport Ability, Perceived Parental Influences and Youths` Enjoyment of Sport and Physical Activity in Botswana. *International Journal of Applied sports sciences (IJASS)*. 2006;18 권 권(2 호 호):89. |  |  |  | X |  |  |
| 67 | Linver MR, Roth JL, Brooks-Gunn J. Patterns of adolescents' participation in organized activities: are sports best when combined with other activities? *Dev Psychol*. Mar 2009;45(2):354-67. |  |  | X |  |  |  |
| 68 | Lubans D, Aguiar EJ, Callister R. The effects of free weights and elastic tubing resistance training on physical self-perception in adolescents. *Psychology of Sport and Exercise*. 2010;11:497-504. |  | X |  |  |  |  |
| 69 | Marsh H. The Effects of Participation in Sport during the Last Two Years of High School. *Sociology of Sport Journal*. 1993;10:18-43. |  |  | X |  |  |  |
| 70 | Martin LJ, Balderson D, Hawkins M, Wilson K, Bruner MW. The influence of social identity on self-worth, commitment, and effort in school-based youth sport. *J Sports Sci*. Feb 2018;36(3):326-332. |  |  |  |  |  | X |
| 71 | Martin LJ, Carron AV, Eys MA, Loughead T. Validation of the Child Sport Cohesion Questionnaire. doi: 10.1080/1091367X.2013.761023. *Measurement in Physical Education and Exercise Science*. 2013/04/01 2013;17(2):105-119. doi:10.1080/1091367x.2013.761023 |  |  |  | X |  |  |
| 72 | McClure AC, Tanski SE, Kingsbury J, Gerrard M, Sargent JD. Characteristics associated with low self-esteem among US adolescents. *Acad Pediatr*. Jul-Aug 2010;10(4):238-44 e2. |  |  |  |  |  | X |
| 73 | McFadden T, Bean C, Fortier M, Post C. Investigating the influence of youth hockey specialization on psychological needs (dis)satisfaction, mental health, and mental illness. doi: 10.1080/23311908.2016.1157975. *Cogent Psychology*. 2016/12/31 2016;3(1):1157975. doi:10.1080/23311908.2016.1157975 |  |  |  | X |  |  |
| 74 | McGale N, McArdle S, Gaffney P. Exploring the effectiveness of an integrated exercise/CBT intervention for young men's mental health. *Br J Health Psychol*. Sep 2011;16(3):457-71. |  |  |  |  |  | X |
| 75 | McMahon EM, Corcoran P, O'Regan G, et al. Physical activity in European adolescents and associations with anxiety, depression and well-being. *Eur Child Adolesc Psychiatry*. Jan 2017;26(1):111-122. |  |  |  |  | X |  |
| 76 | 1Merglen A, Flatz A, Bélanger RE, Michaud PA, Suris JC. Weekly sport practice and adolescent well-being. *Arch Dis Child*. Mar 2014;99(3):208-10. |  |  |  | X |  |  |
| 77 | Michaud PA, Jeannin A, Suris JC. Correlates of extracurricular sport participation among Swiss adolescents. *Eur J Pediatr*. Aug 2006;165(8):546-55. |  |  | X |  |  |  |
| 78 | Moeijes J, van Busschbach JT, Bosscher RJ, Twisk JWR. Sports participation and psychosocial health: a longitudinal observational study in children. *BMC Public Health*. 2018/06/07 2018;18(1):702. doi:10.1186/s12889-018-5624-1 |  |  |  |  |  | X |
| 79 | Moeijes J, van Busschbach JT, Wieringa TH, Kone J, Bosscher RJ, Twisk JWR. Sports participation and health-related quality of life in children: results of a cross-sectional study. *Health and Quality of Life Outcomes*. 2019/04/15 2019;17(1):64. doi:10.1186/s12955-019-1124-y |  |  |  |  |  | X |
| 80 | Mullane SL, Bocchicchio VB, Crespo NC. Feasibility and Parental Acceptability of an 8-Week, Slow-Speed, High-Intensity, Community-Based Resistance Training Program for Preadolescent Children. *Fam Community Health*. Jul/Sep 2017;40(3):183-191. |  | X |  |  |  |  |
| 81 | Newcombe PA, Boyle GJ. High school students' sport personalities: Variations across participation level, gender, type of sport, and success. *International Journal of Sport Psychology*. 1995;26:277-294. |  |  |  | X |  |  |
| 82 | Nixdorf I, Frank R, Beckmann J. Comparison of Athletes’ Proneness to Depressive Symptoms in Individual and Team Sports: Research on Psychological Mediators in Junior Elite Athletes. Original Research. *Frontiers in Psychology*. 2016-June-17 2016;7(893)doi:10.3389/fpsyg.2016.00893 |  |  |  | X |  |  |
| 83 | Ogawa S, Kitagawa Y, Fukushima M, et al. Interactive effect of sleep duration and physical activity on anxiety/depression in adolescents. *Psychiatry Res*. Mar 2019;273:456-460. |  |  |  |  | X |  |
| 84 | Pastor Y, Balaguer I, Pons D, García-Merita M. Testing direct and indirect effects of sports participation on perceived health in Spanish adolescents between 15 and 18 years of age. *J Adolesc*. Dec 2003;26(6):717-30. |  |  |  |  | X |  |
| 85 | Pate RR, Trost SG, Levin S, Dowda M. Sports participation and health-related behaviors among US youth. *Arch Pediatr Adolesc Med*. Sep 2000;154(9):904-11. |  |  |  |  |  | X |
| 86 | Pedersen S, Seidman E. Team Sports Achievement and Self-Esteem Development Among Urban Adolescent Girls. doi: 10.1111/j.1471-6402.2004.00158.x. *Psychology of Women Quarterly*. 2004/12/01 2004;28(4):412-422. doi:10.1111/j.1471-6402.2004.00158.x |  |  | X |  |  |  |
| 87 | 1Perron A, Brendgen M, Vitaro F, Côté SM, Tremblay RE, Boivin M. Moderating effects of team sports participation on the link between peer victimization and mental health problems. *Mental Health and Physical Activity*. 2012/12/01/ 2012;5(2):107-115. doi:https://doi.org/10.1016/j.mhpa.2012.08.006 |  |  |  | X |  |  |
| 88 | Pluhar E, McCracken C, Griffith KL, Christino MA, Sugimoto D, Meehan WP, 3rd. Team Sport Athletes May Be Less Likely To Suffer Anxiety or Depression than Individual Sport Athletes. *J Sports Sci Med*. Sep 2019;18(3):490-496. |  |  |  |  |  | X |
| 89 | Pyle RP, McQuivey RW, Brassington GS, Steiner H. High school student athletes: associations between intensity of participation and health factors. *Clin Pediatr (Phila)*. Oct 2003;42(8):697-701. |  |  | X |  |  |  |
| 90 | Rascle O, Coulomb G, Pfister R. Aggression and goal orientations in handball: influence of institutional sport context. *Percept Mot Skills*. Jun 1998;86(3 Pt 2):1347-60. |  |  |  | X |  |  |
| 91 | Rodriguez D, Moss HB, Audrain-McGovern J. Developmental heterogeneity in adolescent depressive symptoms: associations with smoking behavior. *Psychosom Med*. Mar-Apr 2005;67(2):200-10. | X |  |  |  |  |  |
| 92 | Rodriguez D, Moss HB, Audrain-McGovern J. Developmental heterogeneity in adolescent depressive symptoms: associations with smoking behavior. *Psychosom Med*. Mar-Apr 2005;67(2):200-10. |  |  |  |  |  | X |
| 93 | Sabiston CM, Jewett R, Ashdown-Franks G, et al. Number of Years of Team and Individual Sport Participation During Adolescence and Depressive Symptoms in Early Adulthood. *J Sport Exerc Psychol*. Feb 2016;38(1):105-10. |  |  |  |  | X |  |
| 94 | Sanders CE, Field TM, Diego M, Kaplan M. Moderate involvement in sports is related to lower depression levels among adolescents. *Adolescence*. Winter 2000;35(140):793-7. |  |  | X | X | X |  |
| 95 | Schranz N, Tomkinson G, Parletta N, Petkov J, Olds T. Can resistance training change the strength, body composition and self-concept of overweight and obese adolescent males? A randomised controlled trial. *Br J Sports Med*. Oct 2014;48(20):1482-8. |  | X |  |  |  |  |
| 96 | Seidel RW, Reppucci ND. Organized youth sports and the psychological development of nine-year-old males. *Journal of Child and Family Studies*. 1993/09/01 1993;2(3):229-248. doi:10.1007/bf01321333 |  |  |  | X |  |  |
| 97 | Shields DL, LaVoi NM, Bredemeier BL, Power FC. Predictors of poor sportspersonship in youth sports: personal attitudes and social influences. *J Sport Exerc Psychol*. Dec 2007;29(6):747-62. |  |  |  | X |  |  |
| 98 | Slutzky CB, Simpkins SD. The link between children's sport participation and self-esteem: Exploring the mediating role of sport self-concept. *Psychology of Sport and Exercise*. 2009/05/01/ 2009;10(3):381-389. doi:https://doi.org/10.1016/j.psychsport.2008.09.006 |  |  |  | X |  |  |
| 99 | Snyder AR, Martinez JC, Bay RC, Parsons JT, Sauers EL, Valovich McLeod TC. Health-related quality of life differs between adolescent athletes and adolescent nonathletes. *J Sport Rehabil*. Aug 2010;19(3):237-48. |  |  | X |  |  |  |
| 100 | Steiner H, McQuivey RW, Pavelski R, Pitts T, Kraemer H. Adolescents and Sports: Risk or Benefit? doi: 10.1177/000992280003900304. *Clinical Pediatrics*. 2000/03/01 2000;39(3):161-166. doi:10.1177/000992280003900304 |  |  | X |  |  |  |
| 101 | Steptoe A, Butler N. Sports participation and emotional wellbeing in adolescents. *Lancet*. Jun 29 1996;347(9018):1789-92. |  |  | X |  |  | X |
| 102 | Stiffman AR, Chueh H-j, Earls F. Predictive modeling of change in depressive disorder and counts of depressive symptoms in urban youths. doi:10.1207/s15327795jra0204_1. *Journal of Research on Adolescence*. 1992;2(4):295-316. doi:10.1207/s15327795jra0204_1 | X |  |  |  |  |  |
| 103 | Strachan L, Côté J, Deakin J. “Specializers” versus “samplers” in youth sport: comparing experiences and outcomes. *The sport psychologist*. 2009;23(1):77-92. |  |  |  | X |  |  |
| 104 | Taliaferro LA, Eisenberg ME, Johnson KE, Nelson TF, Neumark-Sztainer D. Sport participation during adolescence and suicide ideation and attempts. *Int J Adolesc Med Health*. 2011;23(1):3-10. |  |  | X |  |  |  |
| 105 | Taliaferro LA, Rienzo BA, Miller MD, Pigg RM, Jr., Dodd VJ. High school youth and suicide risk: exploring protection afforded through physical activity and sport participation. *J Sch Health*. Oct 2008;78(10):545-53. |  |  | X | X |  |  |
| 106 | Thomas GL, Wilson MR. Playing by the Rules: A Developmentally Appropriate Introduction to Rugby Union. doi: 10.1260/1747-9541.10.2-3.413. *International Journal of Sports Science & Coaching*. 2015/06/01 2015;10(2-3):413-423. doi:10.1260/1747-9541.10.2-3.413 |  |  |  | X |  |  |
| 107 | Valois RF, Umstattd MR, Zullig KJ, Paxton RJ. Physical Activity Behaviors and Emotional Self-Efficacy: Is There a Relationship for Adolescents? https://doi.org/10.1111/j.1746-1561.2008.00309.x. *Journal of School Health*. 2008/06/01 2008;78(6):321-327. doi:https://doi.org/10.1111/j.1746-1561.2008.00309.x |  |  | X |  |  | X |
| 108 | Valois RF, Zullig KJ, Huebner ES, Drane JW. Physical activity behaviors and perceived life satisfaction among public high school adolescents. *J Sch Health*. Feb 2004;74(2):59-65. |  |  | X |  |  |  |
| 109 | Van Voorhees BW, Paunesku D, Fogel J, Bell CC. Differences in vulnerability factors for depressive episodes in African American and European American adolescents. *Journal of the National Medical Association*. 2009;101(12):1255-1267. | X |  |  |  |  |  |
| 110 | Velez A, Golem DL, Arent SM. The impact of a 12-week resistance training program on strength, body composition, and self-concept of Hispanic adolescents. *J Strength Cond Res*. Apr 2010;24(4):1065-73. |  | X |  |  |  |  |
| 111 | Vella SA, Cliff DP, Magee CA, Okely AD. Sports participation and parent-reported health-related quality of life in children: longitudinal associations. *J Pediatr*. Jun 2014;164(6):1469-74. |  |  |  | X |  |  |
| 112 | Vella SA, Swann C, Allen MS, Schweickle MJ, Magee CA. Bidirectional Associations between Sport Involvement and Mental Health in Adolescence. *Med Sci Sports Exerc*. Apr 2017;49(4):687-694. |  |  |  |  |  | X |
| 113 | Vink K, Raudsepp L, Kais K. Intrinsic motivation and individual deliberate practice are reciprocally related: Evidence from a longitudinal study of adolescent team sport athletes. *Psychology of sport and Exercise*. 2015;16:1-6. |  |  |  | X |  |  |
| 114 | Wagnsson S, Lindwall M, Gustafsson H. Participation in organized sport and self-esteem across adolescence: the mediating role of perceived sport competence. *Journal of sport and exercise psychology*. 2014;36(6):584-594. |  |  |  | X |  |  |
| 115 | Wang MT, Chow A, Amemiya J. Who Wants to Play? Sport Motivation Trajectories, Sport Participation, and the Development of Depressive Symptoms. *J Youth Adolesc*. Sep 2017;46(9):1982-1998. |  |  |  |  | X |  |
| 116 | Wiersma LD, Fifer AM. “The Schedule Has Been Tough But We Think It's Worth It”: The Joys, Challenges, and Recommendations of Youth Sport Parents. doi: 10.1080/00222216.2008.11950150. *Journal of Leisure Research*. 2008/12/01 2008;40(4):505-530. doi:10.1080/00222216.2008.11950150 |  |  | X |  |  |  |
| 117 | Yang J, Peek-Asa C, Corlette JD, Cheng G, Foster DT, Albright J. Prevalence of and risk factors associated with symptoms of depression in competitive collegiate student athletes. *Clin J Sport Med*. Nov 2007;17(6):481-7. |  |  |  |  |  | X |
| 118 | Zarrett N, Fay K, Li Y, Carrano J, Phelps E, Lerner RM. More than child's play: Variable- and pattern-centered approaches for examining effects of sports participation on youth development. doi:10.1037/a0014577. *Developmental Psychology*. 2009;45(2):368-382. doi:10.1037/a0014577 |  |  | X | X | X |  |
| _aPanza did not mention this in the reference list but it is included as a primary study in their systematic review_ | | | | | | | |

**Amount of primary studies per review:**Cairns, 2014: 15
Collins, 2015: 7
Eime, 2013: 30
Evans, 2017: 35
Panza, 2020: 29
Zuckerman, 2020: 23
Total: 139

**Calculations by Pieper and colleagues ^1^:**
 **1- Percentage of primary studies included in multiple reviews**
Out of the 118 unique primary studies, 17 are included in 2 or more of the 6 reviews. This is a percentage of (7/118)*100% = **14.41%** of the unique primary studies.

**2- Corrected covered Area (CCA)**CCA = (N – r) / ((r*c) – r)
in which N = the number of included publications (including double counting) in evidence synthesis; r = the number of index publications; c = the number of reviews.

CCA = (139-118) / ((118*6) - 118) = **0.0355**

**References:**

1. Pieper D, Antoine SL, Mathes T, Neugebauer EA, Eikermann M. Systematic review finds overlapping reviews were not mentioned in every other overview. *J Clin Epidemiol*. Apr 2014;67(4):368-75. doi:S0895-4356(13)00481-2 [pii] 10.1016/j.jclinepi.2013.11.007
