# Supplementary material for: Impact of organized activities on mental health in children and adolescents: An umbrella review
Source: Prev Med Rep. 2021 Dec 27;25:101687. doi: 10.1016/j.pmedr.2021.101687 (PMC8800068; doi:10.1016/j.pmedr.2021.101687)
Supplement: Supplementary data 3 [file mmc3.docx]

**Appendix C – Formulas**

FORMULA 1 – Odds ratio (OR) to Cohen’s d ^1^

$$d\approx\frac{\sqrt{3}}{\pi}*L_{OR}$$

*with L_OR_ being the natural logarithm of the odds ratio*

FORMULA 2 – Hedge’s $g$ to Cohen’s d ^2^

$$d\approx\frac{1}{J\left( df \right)}*g$$

*with J being the small-sample correction factor*

Or if the sample sizes are large enough:

$$d\approx g$$

FORMULA 3 – Pearson’s r to Cohen’s $d$ ^2^

$$d\approx\frac{2 \cdot r}{\sqrt{1-r^{2}}}$$

**References**

1. Sánchez-Meca J, Marín-Martínez F, Chacón-Moscoso S. Effect-size indices for dichotomized outcomes in meta-analysis. *Psychol Methods*. Dec 2003;8(4):448-67.

2. Fusar-Poli P, Radua J. Ten simple rules for conducting umbrella reviews. *Evidence Based Mental Health*. 2018;21:ebmental-2018. doi:10.1136/ebmental-2018-300014
