# Supplementary material for: Impact of organized activities on mental health in children and adolescents: An umbrella review
Source: Prev Med Rep. 2021 Dec 27;25:101687. doi: 10.1016/j.pmedr.2021.101687 (PMC8800068; doi:10.1016/j.pmedr.2021.101687)
Supplement: Supplementary data 4 [file mmc4.docx]

**Appendix D – Quality of evidence assessment**


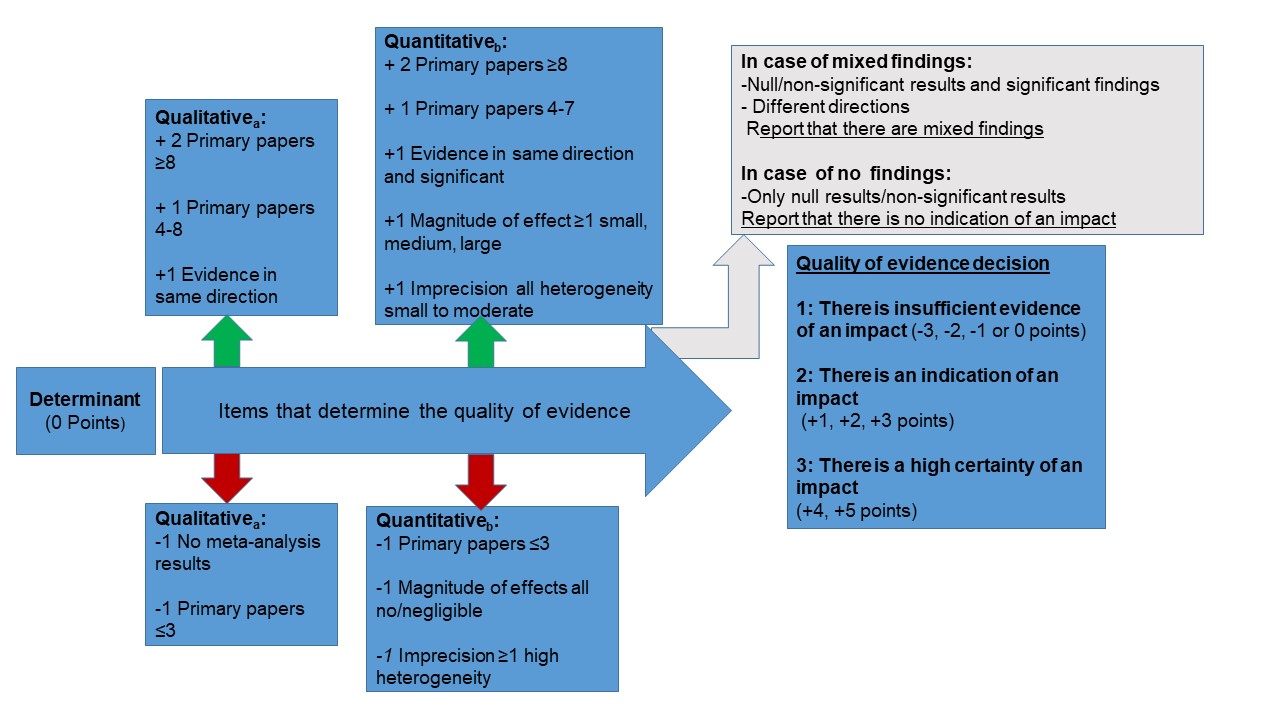


**Figure 1.** Self-developed decision scheme for a quality of evidence assessment for our umbrella review
_a_Qualitative indicates that the results from the systematic reviews to this determinant included no meta-analyses and that we synthesized only qualitative results._b_Quantitative indicates that the results from the systematic reviews to this determinant included meta-analyses and that we synthesized results from the meta-analyses.

| **Table D1.** Self-developed decision scheme and scoring for a quality assessment for our umbrella review | |
| --- | --- |
| **Items that determine the quality of the systematic review** | **points** |
| ***Results from meta-analysis*** |  |
| No | **-1** |
| ***Number of primary papers*** | |
| ≤3 primary papers report on this factor | -1 |
| 4-8 primary papers report on this factor | +1 |
| ≥8 primary papers report on this factor | +2 |
| ***Significance and direction of results*** | |
| *For qualitative results^1^:* All systematic reviews report null results only for this factor  *For quantitative results^2^:* All systematic reviews report non-significant effect sizes for this factor | Stop: report no indication of an influence |
| *For qualitative results_a_:* Systematic reviews report mixed results for this factor   *For quantitative results_b_:*  ≥1 systematic review reports a non-significant effect ≥1 systematic review reports a significant effect size for this factor OR systematic reviews report significant effect sizes in different directions | Stop: report mixed findings |
| *For qualitative results_a_:* All systematic reviews report results in the same direction for this factor  *For quantitative results_b_:* All systematic reviews report significant effect sizes in same direction for this factor | +1 |
| ***Magnitude of effects (NA for qualitative results)*** | |
| All systematic reviews report no or negligible magnitude of effect for this factor | -1 |
| ≥1 systematic review reports a small, medium or large magnitude of effect (i.e. not all report no or negligible magnitude of effect) for this factor | +1 |
| ***Imprecision (NA for qualitative results)*** | |
| ≤1 systematic reviews report high heterogeneity for this factor | -1 |
| All systematic reviews report small to moderate heterogeneity for this factor | +1 |
| NA=not applicable. _a_Qualitative results indicate that the results from the systematic reviews to this determinant included no meta-analyses and that we synthesized only qualitative results. _b_Quantitative results indicate that the results from the systematic reviews to this determinant included meta-analyses and that we synthesized results from the meta-analyses. For systematic reviews the maximum number of points is +3 and minimum number of points is -2. For systematic reviews with meta-analysis the maximum number of points is +5 and the minimum points is -3. Cut-off criteria for quality of evidence decision: 1) -3 points to 0 points indicates insufficient evidence of an impact; 2) +1 points to +3 points indicates that there is evidence of an impact; 3) +4 points to +5 points indicates that there is a high certainty of an impact. In case, of null /non-significant results and significant findings or findings in different directions report that there are mixed findings. In case of only null results/non-significant results report that there is no indication of an impact. | |
