# Supplementary material for: Impact of organized activities on mental health in children and adolescents: An umbrella review
Source: Prev Med Rep. 2021 Dec 27;25:101687. doi: 10.1016/j.pmedr.2021.101687 (PMC8800068; doi:10.1016/j.pmedr.2021.101687)
Supplement: Supplementary data 5 [file mmc5.docx]

**Appendix E – Reference list of excluded papers**

1. **No English Language (n=7)**

- Chacon-Cuberos R, Zurita-Ortega F, Ramirez-Granizo I, Castro-Sanchez M. Physical Activity and Academic Performance in Children and Preadolescents: A Systematic Review. Review. *Apunts Educacion Fisica Y Deportes*. Jan-Mar 2020;(139):1-9.
- Gabrielle Cerqueira da S, Rodrigo Alves dos Santos S, Jorge Lopes Cavalcante N. Saúde mental e níveis de atividade física em crianças: uma revisão sistemática...Mental health and levels of physical activity in children: a systematic review. 2017;25(3):607-615. doi:10.4322/2526-8910.ctoAR0905
- Marti-Vilar M, Gonzalez-Sala F, Dominguez AJ. Prosocial Behaviour in Sports and Physical Activity: A Systematic Review. Review. *Revista Iberoamericana De Psicologia Del Ejercicio Y El Deporte*. 2019;14(2):171-178.
- Seiffer B, Wolf S. Exercise as a possible treatment option in attention deficit hyperactivity disorder. Current study situation and suggestions for specific exercise interventions / Sportliche Aktivitat als mogliche Therapieoption bei ADHS: Aktuelle Studienlage und Vorschlage fur spezifischere Sportinterventionen. *Psychotherapeut*. Jun 2020;65(3):156-159.
- Simoes H, Santos PM, Pereira B, Figueiredo A. Martial Arts and Combat Sports and the Bullying: a systematic review. Review. *Retos-Nuevas Tendencias En Educacion Fisica Deporte Y Recreacion*. 2021;(39):835-843.
- Wang G. Relation between sports activity and education of psychological health in college students. Review. *Chin J Clin Rehab*. 2006;10(24):140-142.
- כרמל ל, מיכל א-ג. האם ההשפעה החיובית של הקראטה על מדדים מוטוריים, קוגניטיביים וחברתיים אצל ילדים טיפוסיים יכולה להוות רציונל לשימוש בקראטה ככלי טיפולי בילדים עם הפרעה התפתחותית בקואורדינציה? *Journal of the Israeli Physical Therapy Society (JIPTS)*. 2019;21(2):40-53.

1. **Other review type (n=14)**

- Arbesman M, Bazyk S, Nochajski SM. Systematic review of occupational therapy and mental health promotion, prevention, and intervention for children and youth. Article. *Am J Occup Ther*. 2013;67(6):e120-e130. doi:10.5014/ajot.2013.008359
- Biddle SJ. Exercise psychology. *Sport Science Review*. 1992;1(2):79-92.
- Bullock GS, Uhan J, Harriss EK, Arden NK, Filbay SR. The Relationship Between Baseball Participation and Health: A Systematic Scoping Review. Article. *J Orthop Sports Phys Ther*. 2020;50(2):55-66. doi:10.2519/jospt.2020.9281
- Cramer H, Lauche R, Dobos G. Characteristics of randomized controlled trials of yoga: a bibliometric analysis. *BMC Altern Med*. Sep 02 2014;14:328.
- Gubbels J, van der Stouwe T, Spruit A, Stams GJJM. Martial arts participation and externalizing behavior in juveniles: A meta-analytic review. Review. *Aggression Violent Behav*. 2016;28:73-81. doi:10.1016/j.avb.2016.03.011
- Gouttebarge V, Bindra A, Blauwet C, et al. International Olympic Committee (IOC) Sport Mental Health Assessment Tool 1 (SMHAT-1) and Sport Mental Health Recognition Tool 1 (SMHRT-1): towards better support of athletes' mental health. Article. *Br J Sports Med*. 2021;55(1):30-37. doi:10.1136/bjsports-2020-102411
- Hansell AH, Giacobbi PR, Voelker DK. A Scoping Review of Sport-Based Health Promotion Interventions With Youth in Africa. Article in Press. *Health Promot Pract*. 2020:1524839920914916. doi:10.1177/1524839920914916
- Johnson KE, Taliaferro LA. Relationships between physical activity and depressive symptoms among middle and older adolescents: A review of the research literature. *Journal for Specialists in Pediatric Nursing*. 2011;16(4):235-251. doi:10.1111/j.1744-6155.2011.00301.x
- Martin J, Liddell M, Roberts S, Greenwood E. Effective therapeutic interventions for Australian adolescents using alcohol and/or other drugs: a scoping review. Review. *Int J Ment Health Syst*. 2020;14(1)doi:10.1186/s13033-020-00425-z
- Rosen L, French A, Sullivan G. Complementary, holistic, and integrative medicine: Yoga. Review. *Pediatr Rev*. 2015;36(10):468-474. doi:10.1542/pir.36-10-468
- Ross G, Nelson R. Using Theater and Drama Interventions to Reduce Bullying in School-Aged Children. *THER RECREATION J*. 2014 4th Quarter 2014;48(4):334-336.
- Spruit A, van Vugt E, van der Put C, van der Stouwe T, Stams GJ. Sports Participation and Juvenile Delinquency: A Meta-Analytic Review. Article. *J Youth Adolesc*. 2016;45(4):655-671. doi:10.1007/s10964-015-0389-7
- Zhang ZJ, Chen WY. A Systematic Review of the Relationship Between Physical Activity and Happiness. Review. *Journal of Happiness Studies*. Apr 2019;20(4):1305-1322.
- Zarobe L, Bungay H. The role of arts activities in developing resilience and mental wellbeing in children and young people a rapid review of the literature. Review. *Perspect Public Health*. 2017;137(6):337-347. doi:10.1177/1757913917712283

1. **Age group not 0-21 year old (n=14)**

- Bridges L, Sharma M. The Efficacy of Yoga as a Form of Treatment for Depression. *J Evid Based Complementary Altern Med*. 10 2017;22(4):1017-1028.
- Grunseit AC, Richards J, Reece L, Bauman A, Merom D. Evidence on the reach and impact of the social physical activity phenomenon parkrun: A scoping review. *Prev Med Rep*. Dec 2020;20:101231.
- Lahart I, Darcy P, Gidlow C, Calogiuri G. The Effects of Green Exercise on Physical and Mental Wellbeing: A Systematic Review. 04 15 2019;
- Letton ME, Thom JM, Ward RE. The Effectiveness of Classical Ballet Training on Health-Related Outcomes: A Systematic Review. Article. *J Phys Act Health*. 2020;17(5):566-574. doi:10.1123/jpah.2019-0303
- Liu X, Clark J, Siskind D, et al. A systematic review and meta-analysis of the effects of Qigong and Tai Chi for depressive symptoms. *Complement Ther Med*. Aug 2015;23(4):516-534.
- Moore B, Dudley D, Woodcock S. The effect of martial arts training on mental health outcomes: A systematic review and meta-analysis. Review. *Journal of Bodywork and Movement Therapies*. Oct 2020;24(4):402-412.
- Nanthakumar C. Yoga for anxiety and depression – a literature review. *Journal of Mental Health Training, Education & Practice*. 2020;15(3):157-169. doi:10.1108/JMHTEP-09-2019-0050
- Noda S, Shirotsuki K, Nakao M. The effectiveness of intervention with board games: A systematic review. *BioPsychoSocial Medicine*. Oct 2019;13:22.
- Sharma M. Yoga as an alternative and complementary approach for stress management: a systematic review. Review. *J Evid Based Complementary Altern Med*. 2014;19(1):59-67. doi:10.1177/2156587213503344
- Sønderlund AL, O'Brien K, Kremer P, et al. The association between sports participation, alcohol use and aggression and violence: A systematic review. Review. *J Sci Med Sport*. 2014;17(1):2-7. doi:10.1016/j.jsams.2013.03.011
- Thompson Coon J, Boddy K, Stein K, Whear R, Barton J, Depledge MH. Does participating in physical activity in outdoor natural environments have a greater effect on physical and mental wellbeing than physical activity indoors? A systematic review. Review. *Environ Sci Technol*. 2011;45(5):1761-1772. doi:10.1021/es102947t
- Williams E, Dingle GA, Clift S. A systematic review of mental health and wellbeing outcomes of group singing for adults with a mental health condition. Article. *Eur J Public Health*. 2018;28(6):1035-1042. doi:10.1093/eurpub/cky115
- Wolpert M, Dalzell K, Ullman R, et al. Strategies not accompanied by a mental health professional to address anxiety and depression in children and young people: a scoping review of range and a systematic review of effectiveness. Review. *Lancet Psychiatry*. 2019;6(1):46-60. doi:10.1016/s2215-0366(18)30465-6
- Yap AF, Kwan YH, Ang SB. A systematic review on the effects of active participation in rhythm-centred music making on different aspects of health. Review. *Eur J Integr Med*. 2017;9:44-49. doi:10.1016/j.eujim.2016.11.011

1. **No general population (n=14)**

- Axelsdóttir B, Biedilæ S, Sagatun Å, Nordheim LV, Larun L. Review: Exercise for depression in children and adolescents – a systematic review and meta-analysis. Article in Press. *Child Adolesc Ment Health*. 2020;doi:10.1111/camh.12438
- Balasubramaniam M, Telles S, Doraiswamy PM. Yoga on our minds: a systematic review of yoga for neuropsychiatric disorders. *Front Psychiatr*. 2012;3:117.
- Cahill SM, Egan BE, Seber J. Activity- and Occupation-Based Interventions to Support Mental Health, Positive Behavior, and Social Participation for Children and Youth: A Systematic Review. Article. *Am J Occup Ther*. 2020;74(2):7402180020p1-7402180020p28. doi:10.5014/ajot.2020.038687
- Cain M, Lakhani A, Istvandity L. Short and long term outcomes for culturally and linguistically diverse (CALD) and at-risk communities in participatory music programs: A systematic review. Review. *Arts Health*. 2016;8(2):105-124.
- Callinan J, Coyne I. Arts-based interventions to promote transition outcomes for young people with long-term conditions: A review. Review. *Chronic Illn*. 2020;16(1):23-40. doi:10.1177/1742395318782370
- Cerrillo-Urbina AJ, García-Hermoso A, Sánchez-López M, Pardo-Guijarro MJ, Santos Gómez JL, Martínez-Vizcaíno V. The effects of physical exercise in children with attention deficit hyperactivity disorder: a systematic review and meta-analysis of randomized control trials. Article. *Child Care Health Dev*. 2015;41(6):779-788. doi:10.1111/cch.12255
- Chimiklis AL, Dahl V, Spears AP, Goss K, Fogarty K, Chacko A. Yoga, Mindfulness, and Meditation Interventions for Youth with ADHD: Systematic Review and Meta-Analysis. Review. *Journal of Child and Family Studies*. Oct 2018;27(10):3155-3168.
- Gauthier A, Kato PM, Bul KC, Dunwell I, Walker-Clarke A, Lameras P. Board games for health: A systematic literature review and meta-analysis. *Games for Health*. Apr 2019;8(2):85-100.
- Harwood A, Lavidor M, Rassovsky Y. Reducing aggression with martial arts: A meta-analysis of child and youth studies. Aggression Violent Behav. 2017;34:96-101.
- James-Palmer A, Anderson EZ, Zucker L, Kofman Y, Daneault JF. Yoga as an Intervention for the Reduction of Symptoms of Anxiety and Depression in Children and Adolescents: A Systematic Review. *Front pediatr*. 2020;8:78.
- Lubans DR, Plotnikoff RC, Lubans NJ. Review: A systematic review of the impact of physical activity programmes on social and emotional well-being in at-risk youth. Review. *Child Adolesc Ment Health*. 2012;17(1):2-13. doi:10.1111/j.1475-3588.2011.00623.x
- Neill RD, Lloyd K, Best P, Tully MA. The effects of interventions with physical activity components on adolescent mental health: Systematic review and meta-analysis. *Mental Health and Physical Activity*. Oct 2020;19:100359.
- Radovic S, Gordon MS, Melvin GA. Should we recommend exercise to adolescents with depressive symptoms? A meta-analysis. Review. *J Paediatr Child Health*. 2017;53(3):214-220. doi:10.1111/jpc.13426
- Riskowski JL, Almeheyawi R. Effects of Tai Chi and Qigong in children and adolescents: A systematic review of trials. *Adolescent Research Review*. Mar 2019;4(1):73-91.

1. **Outcome is not mental health (problems) (n=5)**

- Giraudeau C, Bailly N. Intergenerational programs: What can school-age children and older people expect from them? A systematic review. *Eur j ageing*. Sep 2019;16(3):363-376.
- Hallal PC, Victora CG, Azevedo MR, Wells JC. Adolescent physical activity and health: a systematic review. *Sports Med*. 2006;36(12):1019-1030.
- Kuzik N, Poitras VJ, Tremblay MS, Lee EY, Hunter S, Carson V. Systematic review of the relationships between combinations of movement behaviours and health indicators in the early years (0-4 years). Review. *BMC Public Health*. 2017;17:849. doi:10.1186/s12889-017-4851-1
- Singh AS, Saliasi E, van den Berg V, et al. Effects of physical activity interventions on cognitive and academic performance in children and adolescents: a novel combination of a systematic review and recommendations from an expert panel. Review. *British Journal of Sports Medicine*. May 2019;53(10):640-+.
- Van Hecke L, Loyen A, Verloigne M, et al. Variation in population levels of physical activity in European children and adolescents according to cross-European studies: A systematic literature review within DEDIPAC. Article. *Int J Behav Nutr Phys Act*. 2016;13(1)doi:10.1186/s12966-016-0396-4

1. **Exposure/condition/setting is not organized activities (n=31)**

- Bidzan-Bluma I, Lipowska M. Physical activity and cognitive functioning of children: A systematic review. Review. *Int J Environ Res Public Health*. 2018;15(4)doi:10.3390/ijerph15040800
- Bikomeye JC, Balza J, Beyer KM. The impact of schoolyard greening on children’s physical activity and socioemotional health: A systematic review of experimental studies. Article. *Int J Environ Res Public Health*. 2021;18(2):1-20. doi:10.3390/ijerph18020535
- Birdee GS, Yeh GY, Wayne PM, Phillips RS, Davis RB, Gardiner P. Clinical Applications of Yoga for the Pediatric Population: A Systematic Review. Review. *Acad Pediatr*. 2009;9(4):212-220.e9. doi:10.1016/j.acap.2009.04.002
- Brown HE, Pearson N, Braithwaite RE, Brown WJ, Biddle SJ. Physical activity interventions and depression in children and adolescents : a systematic review and meta-analysis. Review. *Sports Med*. 2013;43(3):195-206. doi:10.1007/s40279-012-0015-8
- Burkhardt J, Brennan C. The effects of recreational dance interventions on the health and well-being of children and young people: A systematic review. *Arts & Health: An International Journal of Research, Policy and Practice*. Jun 2012;4(2):148-161.
- David OA, Costescu C, Cardos R, Mogoase C. How effective are serious games for promoting mental health and health behavioral change in children and adolescents? A systematic review and meta-analysis. *Child & Youth Care Forum*. Dec 2020;49(6):817-838.
- Evans E, Hawton K, Rodham K. Factors associated with suicidal phenomena in adolescents: A systematic review of population-based studies. Review. *Clin Psychol Rev*. 2004;24(8):957-979. doi:10.1016/j.cpr.2004.04.005
- Ferreira-Vorkapic C, Feitoza JM, Marchioro M, Simoes J, Kozasa E, Telles S. Are There Benefits from Teaching Yoga at Schools? A Systematic Review of Randomized Control Trials of Yoga-Based Interventions. *Evid Based Complement Alternat Med*. 2015;2015:345835.
- Glew SG, Simonds LM, Williams EI. The effects of group singing on the wellbeing and psychosocial outcomes of children and young people: a systematic integrative review. Article in Press. *Arts Health*. 2020:1-23. doi:10.1080/17533015.2020.1802604
- Gutierrez-Garcia C, Astrain I, Izquierdo E, Gomez-Alonso MT, Yague JM. Effects of judo participation in children: a systematic review. Review. *Ido Movement for Culture-Journal of Martial Arts Anthropology*. 2018;18(4):63-73.
- Hinkley T, Teychenne M, Downing KL, Ball K, Salmon J, Hesketh KD. Early childhood physical activity, sedentary behaviors and psychosocial well-being: A systematic review. Review. *Prev Med*. 2014;62:182-192. doi:10.1016/j.ypmed.2014.02.007
- Larun L, Nordheim LV, Ekeland E, Hagen KB, Heian F. Exercise in prevention and treatment of anxiety and depression among children and young people. Review. *Cochrane Database Syst Rev*. 2006;3:CD004691.
- Lees C, Hopkins J. Effect of aerobic exercise on cognition, academic achievement, and psychosocial function in children: a systematic review of randomized control trials. Review. *Prev Chronic Dis*. 2013;10:E174. doi:10.5888/pcd10.130010
- Lubans D, Richards J, Hillman C, et al. Physical activity for cognitive and mental health in youth: A systematic review of mechanisms. Review. *Pediatrics*. 2016;138(3)doi:10.1542/peds.2016-1642
- Manferdelli G, La Torre A, Codella R. Outdoor physical activity bears multiple benefits to health and society. Article. *J Sports Med Phys Fitness*. 2019;59(5):868-879. doi:10.23736/s0022-4707.18.08771-6
- Mansfield L, Kay T, Meads C, et al. Sport and dance interventions for healthy young people (15-24 years) to promote subjective well-being: a systematic review. Review. *BMJ Open*. Sep 2018;8(7)
- Mygind L, Kjeldsted E, Hartmeyer R, Mygind E, Bolling M, Bentsen P. Mental, physical and social health benefits of immersive nature-experience for children and adolescents: A systematic review and quality assessment of the evidence. *Health Place*. 07 2019;58:102136.
- Piñeiro-Cossio J, Fernández-Martínez A, Nuviala A, Pérez-Ordás R. Psychological wellbeing in physical education and school sports: A systematic review. Review. *Int J Environ Res Public Health*. 2021;18(3):1-16. doi:10.3390/ijerph18030864
- Rodriguez-Ayllon M, Cadenas-Sánchez C, Estévez-López F, et al. Role of Physical Activity and Sedentary Behavior in the Mental Health of Preschoolers, Children and Adolescents: A Systematic Review and Meta-Analysis. Review. *Sports Med*. 2019;49(9):1383-1410. doi:10.1007/s40279-019-01099-5
- Salazar de Pablo G, De Micheli A, Nieman DH, et al. Universal and selective interventions to promote good mental health in young people: Systematic review and meta-analysis. Review. *Eur Neuropsychopharmacol*. 2020;41:28-39. doi:10.1016/j.euroneuro.2020.10.007
- Schuch FB, Vancampfort D, Firth J, et al. "Physical activity and incident depression: A meta-analysis of prospective cohort studies": Correction. *The American Journal of Psychiatry*. Jun 2018;175(6):574.
- Serwacki ML, Cook-Cottone C. Yoga in the schools: a systematic review of the literature. Review. *Int J Yoga Therap*. 2012;(22):101-109.
- Spruit A, Kavussanu M, Smit T, Ijntema M. The Relationship between Moral Climate of Sports and the Moral Behavior of Young Athletes: A Multilevel Meta-analysis. Article. *J Youth Adolesc*. 2019;48(2):228-242. doi:10.1007/s10964-018-0968-5
- Strong WB, Malina RM, Blimkie CJR, et al. Evidence based physical activity for school-age youth. Review. *J Pediatr*. 2005;146(6):732-737. doi:10.1016/j.jpeds.2005.01.055
- Vekety B, Logemann HNA, Takacs ZK. The effect of mindfulness-based interventions on inattentive and hyperactive–impulsive behavior in childhood: A meta-analysis. Article. *Int J Behav Dev*. 2021;45(2):133-145. doi:10.1177/0165025420958192
- Waddell C, Schwartz C, Andres C, Barican JL, Yung D. Fifty years of preventing and treating childhood behaviour disorders: a systematic review to inform policy and practice. *Evidence Based Mental Health*. 2018;21(2):45-52. doi:10.1136/eb-2017-102862
- Wang M, Sokol R, Luan H, Perron BE, Victor BG, Wu S. Mental health service interventions for left-behind children in mainland China: A systematic review of randomized controlled trials. *Children and Youth Services Review*. Oct 2020;117:105304.
- Weaver LL, Darragh AR. Systematic Review of Yoga Interventions for Anxiety Reduction Among Children and Adolescents. Review. *Am J Occup Ther*. 2015;69(6):p1-p9. doi:10.5014/ajot.2015.020115
- Wegner L, Flisher AJ. Leisure boredom and adolescent risk behaviour: A systematic literature review. *Journal of Child and Adolescent Mental Health*. Jun 2009;21(1):1-28.
- White RL, Babic MJ, Parker PD, Lubans DR, Astell-Burt T, Lonsdale C. Domain-Specific Physical Activity and Mental Health: A Meta-analysis. Review. *Am J Prev Med*. 2017;52(5):653-666. doi:10.1016/j.amepre.2016.12.008
- Yazdi-Feyzabadi V, Mehrolhassani MH, Zolala F, Haghdoost A, Oroomiei N. Determinants of risky sexual practice, drug abuse and alcohol consumption in adolescents in Iran: a systematic literature review. Article. *Reprod Health*. 2019;16(1):115. doi:10.1186/s12978-019-0779-5
